# Muscle-derived factor alleviated cognitive impairment caused by intestinal ischemia-reperfusion

**DOI:** 10.1016/j.redox.2025.103682

**Published:** 2025-05-15

**Authors:** Yafang Tan, Guo Mu, Feixiang Wang, Xin Fan, Chengjie Yang, Zuan Shi, Yiping Bai, Bingqing Xie, Xuan Yu, Jianguo Feng, Jing Jia, Xiaobin Wang, Ye Chen, Jun Zhou

**Affiliations:** a Department of Anesthesiology, The Affiliated Hospital, Southwest Medical University, Luzhou, 646000, China; b Department of Anesthesiology, Zigong Fourth People's Hospital, Zigong, 643000, China; c Anesthesiology and Critical Care Medicine Key Laboratory of Luzhou, Southwest Medical University, Luzhou, 646000, China; d Department of Anesthesiology, Biejing Anzhen Nanchong Hospital of Capital Medical University & Nanchong Central Hospital, Nanchong, 634700, China; e Laboratory of Neurological Diseases and Brain Function, The Affiliated Hospital, Southwest Medical University, Luzhou, 646000, China; f Institute of Epigenetics and Brain Science, Southwest Medical University, Luzhou, 646000, China; g Department of Traditional Chinese Medicine, The Affiliated Hospital, Southwest Medical University, Luzhou, 646000, China

**Keywords:** Muscle, Irisin, Intestinal ischemia reperfusion, Cerebral dysfunctions, Microglia

## Abstract

Intestinal ischemia/reperfusion (II/R) is a common and grave clinical event, with high morbidity and mortality which can cause cerebral dysfunctions. There are no ideal prevention and treatment measures yet. The present study aimed to determine whether muscle-derived factors can alleviate gut-associated cerebral dysfunctions (GACD) following II/R. We measured the tibialis anterior muscle thickness and irisin levels in patients with and without cognitive dysfunction following cardiopulmonary bypass surgery, calculating the correlation between irisin and cognitive impairment. We found that this protective effect is related to muscle-derived irisin. To elucidate the role of irisin in improving GACD, we knocked out FNDC5 to deplete endogenous irisin and supplemented exogenous irisin. Mechanistic insights into irisin's effects on GACD were investigated using *in vivo* and *in vitro* models, incorporating techniques such as transmission electron microscopy, protein docking analysis, gene overexpression, and western blotting. FNDC5/irisin deficiency aggravated cognitive impairments, the pro-inflammation microglia activation, oxidative injury, inflammatory response, neuronal apoptosis and ferroptosis, while recombinant FNDC5/irisin reversed the above changes leading to neurostructural and cognition recovery. Mechanistically, thioredoxin-interacting protein (TXNIP) was activated in the II/R-related neuropathology and was deteriorated in FNDC5/irisin knockout mice. Our results highlight the potential of FNDC5/irisin to slow GACD, providing new insights and potential therapeutic strategies for the prevention and treatment of GACD.

## Highlights

• Intestinal ischemia/reperfusion (II/R) resulted in skeletal muscle damage and cognitive impairment.
• FNDC5/irisin deficiency aggravated II/R-induced cerebral dysfunctions.
• FNDC5/irisin acts on microglia-derived TXNIP to reduce neuroinflammation.
• FNDC5/irisin promoted M2 microglial activation, dampened NLRP3 activation, oxidative stress.

## 1 Introduction

Intestinal ischemia/reperfusion (II/R) is a frequently observed malady with high morbidity and mortality, which usually develop after several clinical situations such as severe burn, hemorrhagic shock, and some surgical procedures [[1], [2], [3], [4]]. It causes intestinal damage and distant organ injury, however, the mechanism underlying the crosstalk between intestine and brain injury remains to be explored [5,6]. Cognitive dysfunction can increase perioperative mortality, reduce patient quality of life and prolong hospitalization. Therefore, it is important to understand how II/R induce gut-associated cerebral dysfunctions (GACD) and obtain suitable prevention and treatment effects.

Our previous studies have found that neuroinflammation caused by abnormal activation of microglia plays an important role in GACD. Reducing the activation of microglia may be a potential strategy for the prevention and treatment of GACD, but the specific targets still need to be further explored [7]. Irisin is a hormone cleaved and secreted by fibronectin type III domain-containing 5 (FNDC5), which is induced by muscles after exercise [[8], [9], [10]]. Accumulating evidence has demonstrated that FNDC5/irisin plays a pivotal role in synaptic plasticity, neurogenesis, and cognitive deficit in neurodegenerative diseases [[11], [12], [13], [14], [15]]. FNDC5/irisin treatment inhibits the activation of microglia in cerebral ischemia-induced neuronal injury [12]. However, no study has yet reported whether FNDC5/irisin changes during II/R and whether it plays a role in the effects of microglia activation on GACD following II/R.

GACD is a remote organ inflammatory injury after intestinal injury. This process is accompanied by the assembly and activation of nucleotide-binding oligomerization domain-like receptor protein 3 (NLRP3) inflammasomes, and causes different degrees of cell damage. Thioredoxin-interacting protein (TXNIP) is an endogenous inhibitor of thioredoxin (TRX) that plays a vital role in maintaining cellular redox balance. Reactive oxygen species (ROS) of different sources promote the dissociation of TRX and TXNIP; subsequently, under oxidative stress, TXNIP dissociates from TRXand directly binds to NLRP3, promoting inflammasome assembly and subsequent caspase-1 activation, resulting in neuroinflammation and neuronal apoptosis [16]. In recent years, TXNIP has been deduced to be involved in microglial activation [17,18], and be related to oxidative stress and neuroinflammation in neurological diseases [[18], [19], [20]]. Previous studies linked oxidative stress to subsequent neuronal death in Parkinson's disease [[21], [22], [23]]. Theoretically, FNDC5/irisin has anti-oxidative stress effect, and TXNIP may be a potential target of FNDC5/irisin. While, whether the TXNIP participates in the II/R-induced microglial activation, and whether it is involved in the roles of FNDC5/irisin in the II/R-induced neuroinflammation, oxidative stress and ferroptosis remain to be elucidated.

Building upon the established link between muscle-derived FNDC5/irisin and central nervous system (CNS) protection, this study specifically investigates its regulatory effects on microglia-mediated neuroinflammation following II/R-induced muscle injury. Through systematic evaluation of neuroinflammatory cascades and microglial activation patterns, we elucidate how FNDC5/irisin modulates NLRP3 inflammasome signaling in microglia to ameliorate subsequent brain alterations and cognitive impairment. These findings not only advance our understanding of the muscle-brain crosstalk in II/R pathology but also establish microglial polarization as a key therapeutic target for irisin-based interventions.

## 2 Materials and methods

### 2.1 Study population

Previous studies have reported a high incidence of II/R in patients undergoing cardiopulmonary bypass (CPB). Mucosal blood flow may be significantly reduced by CPB, mesenteric perfusion may be altered due to primary endothelial dysfunction, and the use of vasoconstrictors during CPB may aggravate this decrease and regardless of the perfusion technique used, all patients will have intestinal mucosal injury accompanied by increased systemic inflammation levels [24], Plasma diamine oxidase (DAO) levels were measured in patients with CPB and healthy volunteers. Serum d-lactic acid (D-LA) and intestinal fatty acid binding protein (iFABP) were measured to evaluate the intestinal injury after CPB. Between January 2022 and January 2023, we conducted a prospective observational study to investigate the causes of cognitive dysfunction associated with II/R in CPB patients. This part of the study was approved by the Ethics Committee of the Fourth People's Hospital of Zigong (ID:2021–041). The study included 33 patients undergoing valvular replacement surgery under CPB. Cognitive dysfunction was identified in 18 patients using the Montreal Cognitive Assessment (MoCA), while the remaining 15 patients showed no cognitive impairment. The control group (*n* = 20) consisted of healthy individuals who underwent routine health examinations at the Zigong Fourth People's Hospital. The inclusion criteria for the study were: (1) age 18–60 years, with an ASA classification of grade II–III; and (2) undergoing first-time single cardiac valve replacement surgery under CPB. Exclusion criteria included: (1) patients with pre-existing psychiatric or behavioral abnormalities; (2) patients with muscle-related disorders; (3) those with malignancies or hematological diseases. Venous blood samples were collected from all patients after entering the operating room. Serum was separated from blood samples, rapidly frozen in liquid nitrogen, and stored at −80 °C. Tibialis anterior muscle thickness was measured using ultrasound probes after blood collection, following a previously established protocol. Patients were divided into cognitive dysfunction (CD) and non-cognitive dysfunction (Non-CD) groups based on postoperative cognitive scores. Correlations were calculated between muscle thickness and serum irisin levels, as well as between irisin levels and cognitive scores.

### 2.2 Animal experiments

This study's animal experiments consisted of four main parts. Part I: By establishing an II/R model, we explored skeletal muscle injury in II/R mice. In addition, we observed brain injury and cognitive changes induced by II/R and preliminarily explored the potential therapeutic role of irisin in GACD. Part II: To determine whether FNDC5/irisin exerts protective effects in GACD, we established an II/R model in *Fndc5*-knockout animals to eliminate irisin. Part III: Based on the findings from the previous parts, we overexpressed TXNIP to investigate the potential mechanism by which FNDC5/irisin ameliorates GACD.

### 2.3 Animal and modeling

Adult male C57BL/6 mice (6–8-weeks-old; body weights of 22–25 g) were purchased from SiPeiFu Experimental Animals Co., Ltd (SiPeiFu, D007, China). *Fndc5* ^*−/−*^ mice on a C57BL/6 background were obtained with CRISPR/Cas9 technology from Cyagen Biosciences Co., Ltd (Guangzhou, China). All animal procedures were approved by the Animal Care Committee of Southwest Medical University, Luzhou, China (approval number: 20220222–002) and conducted in accordance with ARRIVE 2.0 guidelines [25].

The II/R model was developed by following previous protocol [26]. Mice were fasted for 8 h before the processes to establish the II/R model. The animals were anesthetized with isoflurane inhalation, an abdominal midline incision was made, and the superior mesenteric artery was gently exposed and occluded by a micro clip. After 45 min of ischemia, the micro clip was removed, and the abdominal wound was closed by intermittent sutures with 0.25 % ropivacaine infiltrated to reduce postoperative pain irritation. The mice in the Sham group received only isoflurane anesthesia and laparotomy.

### 2.4 Establishment of microglia-depleted mice

PLX5622 is a colony-stimulating factor 1 receptor (CSF1R) inhibitor that efficiently depletes microglia by blocking their survival signaling pathway. The detailed protocol is as follows: PLX5622 (MedChemExpress, HY-114153A, USA) is formulated into standard chow containing 1.2 % sucrose at a concentration of 1200 mg/kg, while the control group receives the same diet without PLX5622. Adult C57BL/6 mice are allowed free access to the PLX5622 diet for 21 d, which results in significant depletion of microglia in the central nervous system.

### 2.5 Cell culture and lentivirus infection

Mouse BV2 microglia and HT22 hippocampal neuronal cell lines were purchased from American Type Culture Collection and cultured in Dulbecco's modified eagle medium (Gibco by Life Technolohies, 30030, NY) supplemented with 10 % fetal bovine serum (VivaCell, C04001-050, China) and 1 % penicillin/streptomycin antibiotic at 37 °C in a 5 % CO_2_ incubator. The BV2 cells were treated with lipopolysaccharide 1 μg/mL (LPS; Sigma, 0127: B8, UK) for 24 h; then the medium was changed, and the cells were incubated for an additional 6 h. Subsequently, the supernatant was collected and added to HT22 cells for 12 h. BV2 microglia cells were transduced with TXNIP-overexpressing lentiviral vectors (LV-TXNIP-EGFP, HanBio Technology, China). The construct contained full-length mouse TXNIP cDNA (NCBI Accession No. NM_001009935.2) driven by a CMV promoter, with EGFP as a reporter. Viruses were purified at 1 × 10^8^ TU/mL and used at an MOI of 50 in the presence of 5 μg/mL polybrene. Control cells received empty vector (LV-EGFP, HanBio). Transduction efficiency (>85 %) was confirmed by fluorescence microscopy (EGFP^+^) and TXNIP expression was validated by quantitative reverse transcription polymerase chain reaction (qRT-PCR).

### 2.6 Morris water maze (MWM) test

The MWM test evaluated the spatial learning capacity and reference memory as reported previously [7]. Before II/R, the mice were trained for 5 consecutive days, four times a day, to memorize the location of a platform. During each experiment, the mice were placed on a different start quadrant and allowed to find a platform submerged 1 cm below the water surface within a maximum time of 60 s; the escape latency was recorded. Animals that failed to locate the platform within 60 s were manually guided to the platform. The II/R was conducted on day 5 after MWM training, and the 60-s probe test was performed 48 h after II/R. In the probe trial, the platform was removed from the water, and the mice were allowed to swim freely in the water again for 60 s to test their spatial memory. The swimming track, the speed of the mice, and the times of platform crossing were monitored and recorded (XinRuan Information Technology, Smart V3.0, China).

### 2.7 Y-maze test

The Y-maze test was conducted to evaluate spatial working memory and exploratory behavior in mice. The apparatus consisted of three arms (labeled A, B, and C) of equal dimensions (30 cm in length, 8 cm in width, and 15 cm in height) arranged at a 120° angle from one another. The experiment was performed in two phases: Training phase: Each mouse was placed at the center of the maze and allowed to explore only two arms (A and B) for 5 min, while the third arm (C) was blocked by a removable barrier. During this phase, the number of entries and time spent in each accessible arm were recorded to confirm normal exploratory behavior and exclude any pre-existing bias toward a specific arm. Testing phase: After a 1-h intertrial interval, the barrier to arm C was removed, allowing the mouse to explore all three arms freely for 5 min. The total number of entries into each arm and the time spent in the novel arm (C) were recorded. The percentage of time spent in the novel arm and the percentage of entries into the novel arm were calculated as indices of spatial memory and exploratory drive.

### 2.8 DNA extraction and genotyping

Tail DNA samples were extracted from 2-week-old FNDC5/irisin−/− mice, following instructions of the DNA Quick Extraction Kit (Beyotim, D0065S, China). Standard PCR was performed on all the generated mice. Mice were grouped based on the genotyping results.

### 2.9 Drug administration

A volume of 100 μl vehicle (0.9 % saline) or recombinant FNDC5/irisin (Peprotech, 100-65-50 μg, USA) was administered at three different doses (1 μg/kg, 5 μg/kg, and 10 μg/kg) by bolus injection via the tail vein immediately after intestinal ischemia [26]. The adeno-associated viruses (1 × 10^12^ virus genome/mL) were obtained from Hesheng Gene Technology Co., Ltd (Shenzhen, China). About 4 weeks before inducing II/R, 3-4-week-old male C57BL/6 mice were anesthetized by Nembutal, i. p. 0.7 mg/kg and 2 μL AAV-TXNIP-EGFP or AAV-EGFP viral particles were stereotactically injected into the bilateral CA1 regions of the hippocampus at a rate of 0.5 μL/min with a Hamilton syringe using a micropump. The needle was left in place for 5 min and then raised over slowly.

### 2.10 Histological analysis

4-μm-thick hippocampus and intestine sections were de-paraffinized, stained with H&E, and observed under a light microscope (Olympus, Tokyo, Japan). The histopathological analyses of intestinal mucosa were performed using a modified Chiu's score by two experienced pathologists blinded to the study [27]. The number of normal neurons per unit area was determined by randomly sampling under four high-power visual fields of the CA1 region of the hippocampus.

### 2.11 Detection of inflammatory factors

Hippocampal homogenates were prepared at low temperature. The supernatants were collected by centrifugation at 4 °C and 13000 rpm for 20 min. The levels of IL-1β, TNF-α, and IL-6 in the plasma, hippocampus, and BV2 cells were detected by the ELISA kit, according to the manufacturer's instructions (Lianke, IL-1β: EK201BHS, TNF-α: EK282HS, IL-6: EK206, China).

### 2.12 Detection of lipid peroxidation, glutathione (GSH), and ferrous ion (Fe^2+^)

*In vivo,* lipid oxidation was evaluated by measuring thiobarbituric acid reactive substances (TBARS) with a commercial kit (Meimian Biotechnology, MM-0897M1, China) following the manufacturer's protocol. The formed colored adduct were quantified at 532 nm. Glutathione (GSH) levels were determined via the 5,5′-dithiobis-2-nitrobenzoic acid (DTNB) assay, measuring thiol-group conjugation at 412 nm with a commercial kit (Elabscience, E-BC-K030-M, USA). Fe^2+^ content was analyzed using a ferrozine-based colorimetric kit, where Fe^3+^ is reduced to Fe^2+^ and complexed with ferrozine (absorbance at 562 nm), which was conducted using the kits according to the manufacturer's instructions (Meimian Biotechnology, MM-46281M1, China). *In vitro*, the intracellular oxidants react with dihydroethidium (DHE, Yeasen Biotechnology, 50102ES25, China). HT22 cells were incubated with 10 μmoL DHE at 37 °C for 60 min in a humidified and dark chamber and then counterstained with 4′,6-diamidino-2-phenylindole (DAPI, Solarbio, C0065, China). The relative fluorescence intensity was measured using Image J software (National Institutes of Health, Bethesda, MD) and normalized to the cell number. GSH and Fe^2+^ was measured to determine iron deposits in the hippocampal neurons.

### 2.13 Wheat germ agglutinin (WGA) staining

After baking paraffin sections at 55 °C for 30 min, immerse them sequentially in xylene (3 × 5 min), absolute ethanol (5 min), 95 % ethanol (5 min), and 70 % ethanol (5 min), followed by rinsing in distilled water (3 × 5 min). For antigen retrieval, incubate the sections in preheated citrate buffer (pH 6.0) at 95 °C for 30 min, then allow them to cool naturally to room temperature before rinsing under running water (3 × 5 min). Next, apply 30 μL of iF555-Wheat Germ Agglutinin (1:200, Servicebio, G1731-100UL, China) to each section and incubate at 37 °C in a dark humidified chamber for 30 min. After washing with PBST (3 × 5 min), counterstain the nuclei with ready-to-use DAPI for 10 min, followed by another PBST wash (3 × 5 min). Briefly dry the sections, then mount them with antifade mounting medium and coverslips. Finally, observe the slides under a fluorescence microscope, acquire images, and analyze them using Image-Pro Plus software.

### 2.14 Non-invasive compound muscle action potential (CMAP)

Under anesthesia, the mouse was placed in a prone position, and the femoral nerve was exposed near the inguinal region. A bipolar stimulating electrode was carefully positioned around the nerve, while a recording electrode was inserted into the quadriceps muscle belly. A reference electrode was placed subcutaneously nearby. Square-wave pulses (0.1 ms duration, 0.5–5 mA intensity) were delivered using an isolated stimulator to evoke CMAPs. Signals were amplified ( × 1000), bandpass-filtered (10 Hz–10 kHz), and digitized at 50 kHz using a data acquisition system. Stimulation intensity was gradually increased until CMAP amplitude plateau. Latency was measured from stimulus onset to the first negative deflection, and amplitude was calculated peak-to-peak. Nerve conduction velocity was determined by dividing the distance between stimulating and recording sites by the latency. Data were averaged over 3–5 trials. After recording, the wound was sutured, and the mouse was allowed to recover on a warming pad.

### 2.15 Skeletal muscle fatigue assessment

Freshly isolated mouse skeletal muscles (quadriceps femoris) were mounted in a tissue bath containing oxygenated (95 % O_2_/5 % CO_2_) Ringer's solution (37 °C, pH 7.4). Muscles were adjusted to optimal length and preloaded with 1–2 g resting tension. After determining maximal tetanic force via electrical stimulation (100 Hz, 0.5 ms pulses, 10–20 V), fatigue was induced by repeated tetanic contractions (1 train/sec, 300 ms duration, 100 Hz frequency) for 5 min. Force production was continuously recorded. The fatigue index was calculated as (force at 5 min/initial maximal force) × 100 %. Additional analysis included time to 50 % force reduction and rate of force decline. Between experiments, solution was refreshed every 15 min to maintain pH and metabolite concentrations.

### 2.16 Transmission electron microscope (TEM) imaging

Fresh hippocampal tissues were rapidly dissected and fixed in 2.5 % glutaraldehyde (4 °C, 24 h), then post-fixed with 1 % osmium tetroxide (2 h). After dehydration through graded ethanol series (50–100 %), samples were embedded in epoxy resin and polymerized (60 °C, 48 h). Ultrathin sections (70 nm) were cut using a diamond knife (Leica UC7), stained with uranyl acetate/lead citrate, and imaged by TEM (Hitachi HT7800, 80 kV).

### 2.17 Terminal deoxynucleotidyl transferase dUTP nick-end labeling (TUNEL) staining

Apoptosis of the hippocampus and HT22 cells was detected using In Situ Apoptosis Detection Kit (Roche, 11684817910, Switzerland) The coronal cryosections for the detection of neuronal apoptosis were stained with a neuron marker (NeuN) and TUNEL staining, and HT22 cell slides were stained with TUNEL staining. The cryosections were processed for NeuN (1; 200, Abcam, ab104224, UK).

### 2.18 Immunofluorescence staining

The coronal cryosections of the brain and BV2 cell slides were fixed in paraformaldehyde for 30 min, permeabilized with 0.2 % Triton X-100 for 15 min, and blocked in 1 % bovine serum albumin for 30 min at room temperature. Then, the sections were incubated at 4 °C overnight with the following primary antibodies: anti-TXNIP (1:200, Affinity, DF7506, China), anti-CD16 (1:50, Santa Cruz Biotechnology, sc-58962, CA), or anti-CD206 (1:50, Santa Cruz Biotechnology, sc-34577, CA). After an additional 10-min fixation with 4 % paraformaldehyde, a second stained step with anti-NeuN (1; 200, Abcam, ab104224, UK), anti-GFAP (1:200, CST, 3670s, USA), anti-Iba-1 (1:200, Abcam, AB178846, UK), or anti-Iba-1 (1:200, CST, 17198S, USA) was effectuated at room temperature for 1 h. Finally, the sections were washed with 0.01 M of PBS and incubated with fluorescence-conjugated secondary antibodies at room temperature for 1 h. After washing, the slides were sealed with 50 % glycerol and an anti-quenching agent and observed under a fluorescence microscope. The relative fluorescence intensity was determined using Image J and was normalized to the cell number.

### 2.19 Western blot analysis

Hippocampal tissues or BV2 cells were lysed using radioimmunoprecipitation assay (RIPA) buffer supplemented with 1 % phenylmethylsulfonyl fluoride (PMSF). Protein concentrations were determined using the Bradford method with bicinchoninic acid assay (BCA) standards. Samples (30 μg/lane) were separated by SDS-PAGE (7.5–12.5 %) and transferred to nitrocellulose membranes. After blocking with 5 % non-fat milk, membranes were incubated overnight at 4 °C with primary antibodies: FNDC5 (Abcam, ab174833, UK), TXNIP (CST, 14715s, USA), NLRP3 (CST, 15101s, USA), Caspase-1 (Santa Cruz Biotechnology, sc56036, CA), Caspase-3 (CST, 9661, USA), Bcl-2 (CST, 4223, USA), GPX4 (CST, 93790, USA), Bax (Proteintech, 50599-2-Ig China), iNOS (Affinity, AF0199 China), Arg-1 (Affinity, DF6657, China) and β-actin (Proteintech, 66009-1-Ig, China). Negative controls omitted primary antibodies. After tris-buffered saline with tween® 20 (TBST) washes, membranes were incubated with HRP-conjugated secondary antibodies for 1 h at room temperature. Bands were visualized by enhanced chemiluminescence and quantified using ImageJ.

### 2.20 qRT-PCR

Total RNA was extracted from the hippocampal tissues or BV2 cells using TRIzol reagent (Invitrogen, 15596026, USA). Reverse transcription was conducted using the HiScript III All-in-one RT SuperMix Perfect for qPCR (Vazyme Biotech, R333-01, China), according to the manufacturer's protocol. qRT-PCR was performed using SuperReal PreMix Plus-SYBR Green (Vazyme Biotech, Q221-01, China) on a real-time PCR system (Roche, IN, USA). The PCR amplification parameters were as follows: 95 °C for 30 s, followed by 35 cycles of 95 °C for 10 s and 60 °C for 30 s, and finally, 95 °C for 15 s, 60 °C for 60 s, and 95 °C for 15 s. GAPDH was used as an internal reference for the quantification of TXNIP gene expression level. The normalized mRNA expression level in the naive group (target mRNA/GAPDH value) was utilized to calculate the fold-changes of the mRNA levels in the other groups. Biological replicates were six samples and two technical replicates were performed. The primer sequences are shown in Supplementary 1 Table S1.

### 2.21 Protein interaction analysis

The protein structures of FNDC5, NLRP3 and TXNIP were downloaded from Uniprot database (www.uniprot.org), and the interaction mode between FNDC5 and NLRP3, FNDC5 and TXNIP was studied by Hdock (Huazhong University of Science and Technology, China) and Pymol 2.3.0 (Schrö, USA) is used to analyze the interaction mode of the docking result.

### 2.22 Co-immunoprecipitation (Co-IP) assay

To investigate protein interactions, BV2 cells were lysed in NP-40 buffer containing protease inhibitors. For each reaction, 500 μg of total protein was incubated with 2 μg anti-irisin antibody (Affinity, DF13019, China) or IgG control (Proteintech, 30000-0-AP, China) overnight at 4 °C with gentle rotation. Protein A/G magnetic beads (MedChemExpress, HY-K0202, USA) were then added for 2 h. After washing 3 × with lysis buffer, bound proteins were eluted in 2 × SDS loading buffer at 95 °C for 10 min. Lysates were alternatively precipitated with anti-TXNIP or anti-NLRP antibodies. Eluates were analyzed by Western blot using antibodies against irisin (1:1000, Affinity DF13019, China), TXNIP (1:1000, CST, 14715s, USA), and NLRP3 (1:800, Santa Cruz, sc-66846, CA).

### 2.23 Measurement of nitric oxide (NO) production

BV2 cells were seeded into 96-well plates at a density of 2 × 10^5^ cells/mL and cultured overnight. After 24 h of treatment with LPS (1 μg/mL) alone or LPS (1 μg/mL) plus recombinant FNDC5/irisin (5 nM), the culture supernatants were collected, and the NO production was determined using the NO Content Assay kit (Solarbio, BC1475, China).

### 2.24 Cell viability assay

HT22 cells were plated at a density of 2 × 10^4^ cells/well in a 96-well plate, treated with the conditioned medium (CM) of BV2 cells, and incubated for 12 h. Subsequently, CCK-8 (10 μL) reagent was added to each well of the 96-well plate, and the plate was incubated at 37 °C for 1 h. The viable cells were counted by absorbance measurements at 450 nm. The data were presented as the mean values of three replicate experiments, each performed in triplicate.

### 2.25 Flow cytometry

For flow cytometry, HT22 cells were seeded at a density of 150 × 10^4^ cells/well in a 6-well plate and treated with CM of BV2 cells for 12 h. Then, the cells were collected and washed twice with cold PBS. Finally, the cells were stained using a PE Annexin V Apoptosis Detection Kit (BD Biosciences, 559763, CA) according to the manufacturer's instructions [28].

### 2.26 Statistical analysis

Data were presented as the mean ± standard error of the mean (SEM) and analyzed using GraphPad Prism 8.3 statistical software (San Diego, CA). Linear regression analysis was used for correlation analysis. The statistical analysis of the data was carried out using analysis of variance (ANOVA), followed by Tukey's multiple-comparison post hoc test. For the training phase of the MWM test, the escape latency over a period was analyzed by two-way repeated-measures ANOVA [27]. Kaplan–Meier survival curves were analyzed using the log-rank test. *P* < 0.05 indicated a statistically significant difference.

## 3 Results

### 3.1 II/R leads to skeletal muscle damage

**Fig. 1:**
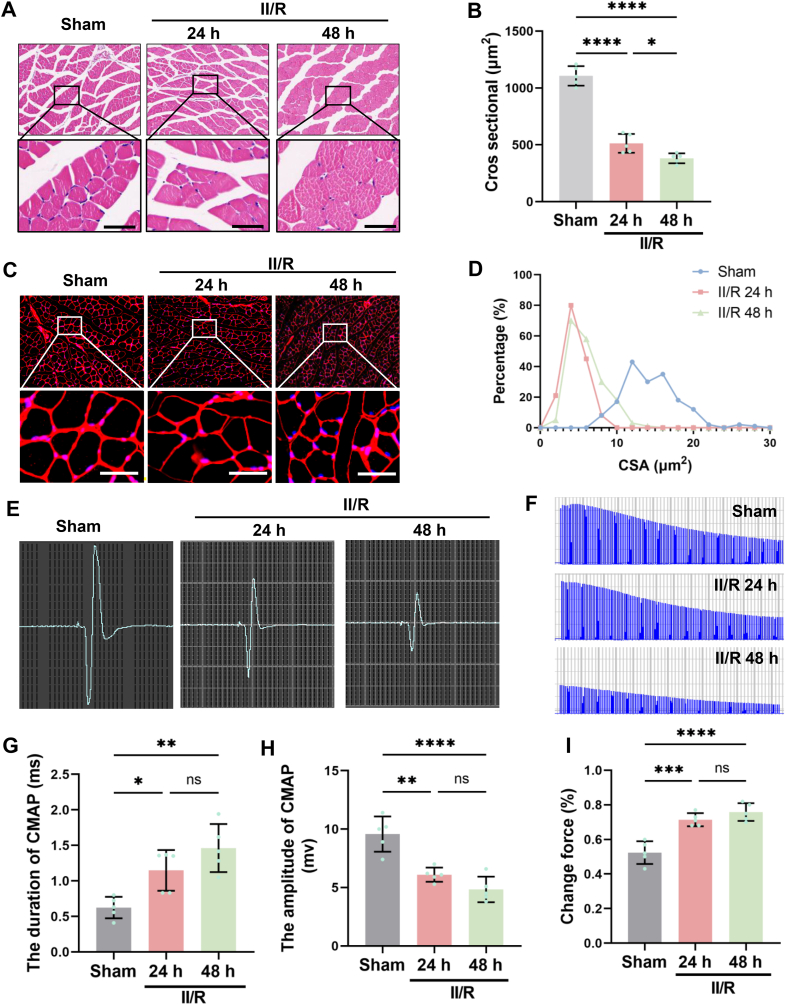
II/R causes skeletal muscle damage. **(A)** Representative H&E staining images of skeletal muscle. Scale bar = 100 μm. **(B)** Quantitative analysis of skeletal muscle fiber cross-sectional area. **(C)** Representative WGA staining images of skeletal muscle. Scale bar = 100 μm. **(D)** Distribution curve of individual muscle fiber cross-sectional areas. **(E)** Representative compound muscle action potential (CMAP) waveforms. **(F)** Ex vivo muscle fatigue curves induced by electrical stimulation. **(G)** Statistical analysis of CMAP latency. **(H)** Statistical analysis of CMAP amplitude. **(I)** Quantitative analysis of muscle fatigue index. (Data are presented as mean ± SD; *n* = 5, ^*ns*^*P*>0.05, ∗*P* < 0.05, ∗∗*P* < 0.01 ∗∗∗*P* < 0.001).

By establishing an II/R model, we investigated alterations in murine skeletal muscle. The results demonstrated that I/R mice began to exhibit reduced muscle fiber diameter at 24 h post-operation. To exclude potential confounding effects of dehydration, we further examined skeletal muscle changes at 48 h post-operation, which revealed a significant decrease in cross-sectional area of muscle fibers in II/R mice (Fig. 1A–D). Subsequent functional assessments showed that II/R injury significantly prolonged CMAP latency while markedly reducing amplitude, indicating impaired neuromuscular transmission. Furthermore, fatigue index measurements demonstrated significantly diminished anti-fatigue capacity in II/R mice (Fig. 1E–I).

### 3.2 Preoperative plasma irisin level is associated with postoperative cognitive impairment in patients undergoing CPB

**Fig. 2:**
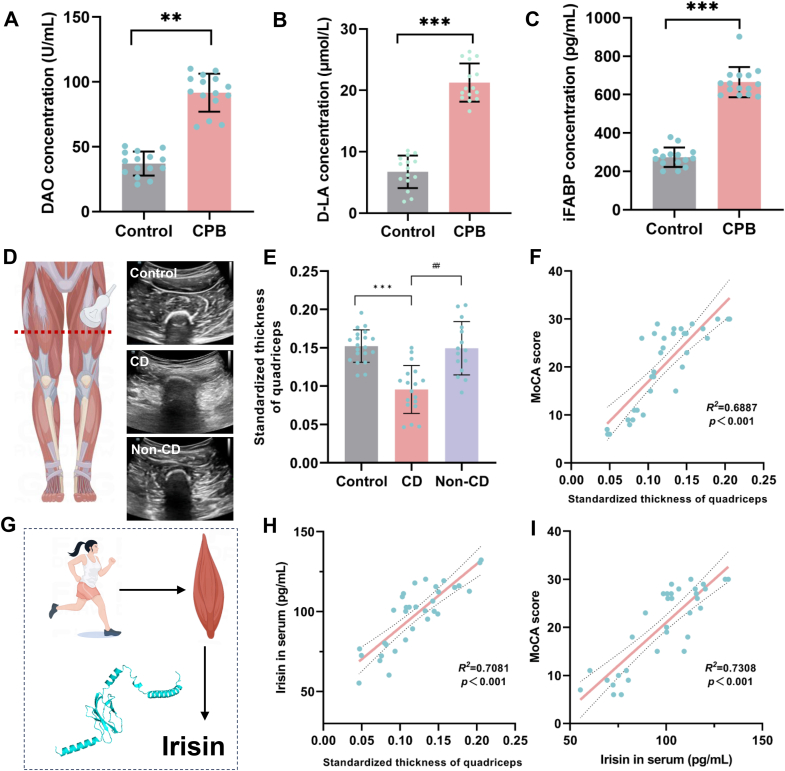
Correlation between quadriceps thickness and irisin and postoperative cognitive ability in CPB patients. **(A)** Plasma DAO levels in CPB patients. **(B)** Plasma D-LA levels in CPB patients. **(C)** Plasma iFABP levels in CPB patients. **(D)** Location of ultrasound scan and representative ultrasound images. **(E)** Standardized thickness of quadriceps of the three groups. **(F)** correlation between standardized quadriceps thickness and MoCA score. **(G)** Schematic representation of irisin production. **(H)** Correlation between plasma irisin levels and quadriceps thickness. **(I)** Correlation between plasma irisin levels and MoCA scores. (Data are presented as mean ± SD; *∗∗P* < 0.01, *∗∗∗P* < 0.001 *vs.* Control group; ^*##*^*P* < 0.01 *vs.* CD group).

To further investigate the role of muscular factors in brain injury induced by II/R, we assessed changes in plasma irisin levels in CPB patients. First, we validated the presence of intestinal injury in the CPB cohort. Plasma levels of DAO, D-LA and iFABP were significantly increased in CPB patients, indicating the presence of II/R (Fig. 2A–C). We measured the preoperative thickness of the tibialis anterior muscle and plasma irisin levels in patients undergoing CPB. A total of 45 CPB patients were recruited, among whom 33 met the inclusion and exclusion criteria and underwent postoperative cognitive assessment using the MoCA-scale. Based on a MoCA score threshold of 26, patients were divided into two groups: those with cognitive impairment (*n* = 18) and those without cognitive impairment (*n* = 15), The inclusion and exclusion process is shown in Supplementary material 2 Fig. S1. Subsequent analysis revealed that the tibialis anterior muscle thickness was significantly lower in patients with cognitive impairment compared to those without (Fig. 2D and E). Additionally, cognitive scores were strongly and positively correlated with tibialis anterior muscle thickness (*R*^*2*^ = 0.6887, *P* < 0.001; Fig. 2F). Given that irisin is predominantly secreted by skeletal muscle (Fig. 2G) and that tibialis anterior muscle thickness was associated with cognitive function in CPB patients, we hypothesized that differences in plasma irisin levels might explain the variability in cognitive impairment among these patients. To test this hypothesis, we analyzed the correlation between plasma irisin levels and tibialis anterior muscle thickness. The results demonstrated that greater muscle thickness was associated with higher plasma irisin levels (*R*^*2*^ = 0.7081, *P* < 0.001; Fig. 2H). Further analysis showed a strong positive correlation between irisin levels and cognitive scores (*R*^*2*^ = 0.7308, *P* < 0.001; Fig. 2I).

### 3.3 II/R leads to brain damage and cognitive impairment, while irisin has the potential to reverse this damage

**Fig. 3:**
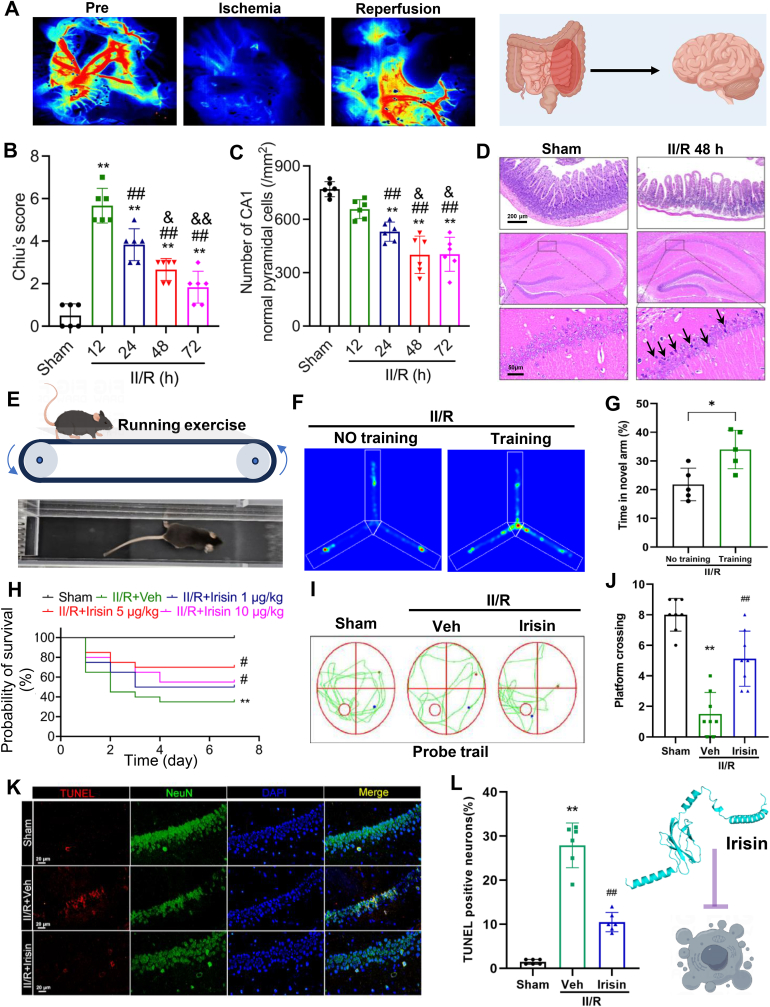
Potential neuroprotective effects of FNDC5/irisin in II/R-induced brain injury. **(A)** Establishment of the mouse model for intestinal ischemia/reperfusion (II/R) injury, with representative laser speckle contrast imaging (LSCI) of mesenteric blood flow. **(B)** Intestinal injury evaluated using Chiu's scoring system (Data are represented as mean ± SD; *n* = 6; *∗∗P* < 0.01 *vs.* Sham group; ^*##*^*P* < 0.01 *vs.* II/R 12 h group; ^*&*^*P* < 0.05,^*&&*^*P* < 0.01 *vs.* II/R 24 h group). **(C)** Quantification of normal neurons in the CA1 region of the hippocampus (Data are represented as mean ± SD; *n* = 6; ^*##*^*P* < 0.01 *vs.* II/R 12 h group; ^*&*^*P* < 0.05 *vs.* II/R 24 h group). **(D)** Representative H&E staining of intestinal tissue (*n* = 6, scale bars = 200 μm) and the CA1 region of the hippocampus (*n* = 6, scale bars = 50 μm) at 48 h post-II/R injury, black arrows indicate cells with damage. **(E)** Mouse treadmill training protocol. **(F**–**G)** Representative heatmaps of Y-maze spontaneous alternation test trajectories and the percentage of new arm entries in II/R-injured mice subjected to treadmill training versus non-trained controls at 48 h post-II/R (*n* = 5; Data are represented as mean ± SD;*∗P* < 0.05 *vs.* No training group). **(H)** Effect of different doses of recombinant FNDC5/irisin on the 7-day survival rate of wild-type (WT) mice following II/R injury (*n* = 20). **(I**–**J)** Representative swimming trajectories in the MWM test and the number of platform crossings during the probe trial (*n* = 8). **(K)** Representative TUNEL staining images showing apoptotic neurons in the CA1 region of the hippocampus after recombinant FNDC5/irisin treatment in II/R-injured mice (Data are represented as mean ± SD; *n* = 6; scale bars = 20 μm; ∗∗*P* < 0.01 *vs*. Sham group; ^##^*P* < 0.01 *vs*. Veh group). **(L)** Quantification of relative fluorescence intensity of TUNEL-positive cells in the CA1 region (*n* = 6; Scale bars = 20 μm; Data are represented as mean ± SD; ∗∗*P* < 0.01 *vs*. Sham group; ^##^*P* < 0.01 *vs*. Veh group).

Using a model of II/R, we investigated whether II/R induces remote brain injury (Fig. 3A). Histopathological examination revealed that intestinal damage peaked at 12 h post-reperfusion, after which intestinal mucosal injury gradually recovered over time. However, injury in the distal brain, particularly in the hippocampal CA1 region, progressively worsened, reaching its peak at 48 h post-reperfusion and subsequently stabilizing (Fig. 3B–D).

Clinical observations suggested that plasma irisin levels were associated with postoperative cognitive function. To explore this relationship, we elevated plasma irisin levels in wild-type (WT) mice through a 5-day regimen of treadmill training. Post II/R injury, these trained mice exhibited significantly better short-term memory performance compared to non-trained controls (Fig. 3E–G). These findings suggest that irisin may have a potential role in mitigating brain injury following II/R. To further investigate, we treated II/R-injured mice with varying doses of irisin. Results demonstrated that intravenous administration of irisin significantly improved the survival rate of II/R mice at all tested doses, with the 5 μg/kg dose providing the most pronounced protective effect (Fig. 3H). Moreover, this dose notably enhanced spatial memory performance in II/R mice (Fig. 3I and J) and significantly reduced hippocampal neuronal apoptosis, we used the neuron-specific marker molecule NeuN to display the neuron location (Fig. 3K and L).

### 3.4 FNDC5/irisin deficiency aggravated II/R-induced brain alteration and cognitive deficit

**Fig. 4:**
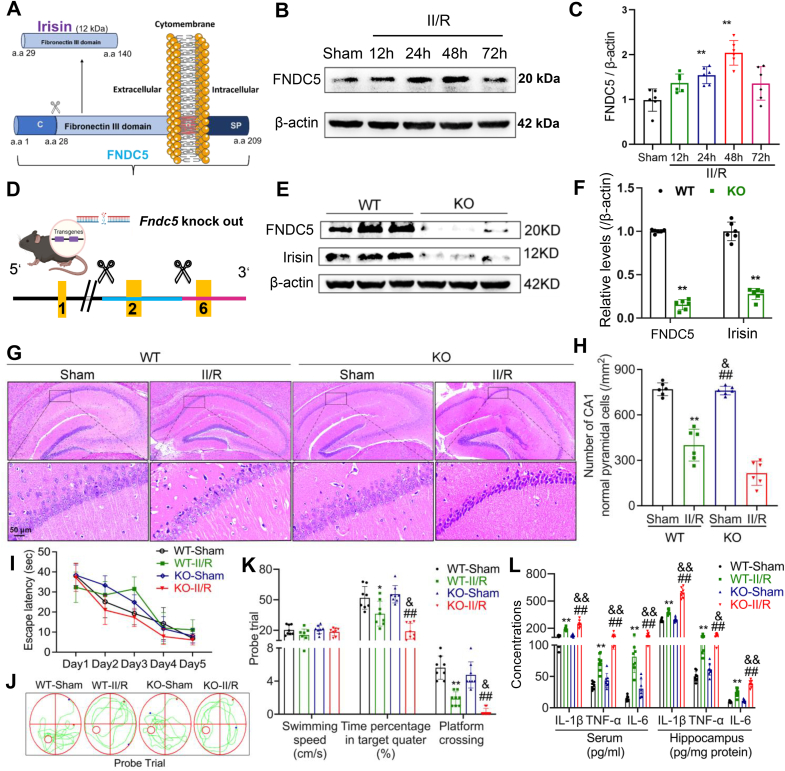
Knockout of FNDC5/irisin exacerbates brain injury following II/R. **(A)** Schematic representation of FNDC5 cleavage to generate irisin. **(B)** Representative WB bands showing FNDC5 levels at different time points post-II/R injury and β-actin was used as loading control. **(C)** Relative levels of FNDC5 (Data are presented as mean ± SD; *n* = 6; *∗∗P* < 0.01 *vs.* Sham group). **(D)** Schematic diagram of the *Fndc5* gene knockout locus. **(E)** Representative WB bands confirming the loss of FNDC5 and irisin relative levels after FNDC5 knockout, β-actin was used as loading control, FNDC5 and irisin correspond to separate loading controls. **(F)** Relative levels of FNDC5 and irisin (Data are presented as mean ± SD; *n* = 6; *∗∗P* < 0.01 *vs.* WT group). **(G)** Representative H&E staining of the CA1 region of the hippocampus in *Fndc5* knockout mice at 48 h post-II/R injury (*n* = 6, scale bars = 50 μm). **(H)** Quantification of normal neurons in the CA1 region of the hippocampus (Data are presented as mean ± SD; *n* = 6; *∗∗P* < 0.01 *vs.* WT-Sham group; ^*##*^*P* < 0.01 *vs.* KO-Sham group; ^*&*^*P* < 0.05 *vs.* WT-II/R group). **(I–K)** MWM test results, including escape latency, time spent in the target quadrant, and platform crossings (*n* = 8; Data are presented as mean ± SD; *∗P* < 0.05, *∗∗P* < 0.01 vs. WT-Sham group; ^*##*^*P* < 0.01 vs. KO-Sham group; ^*&*^*P* < 0.05 vs. WT-II/R group). **(L)** concentration of inflammatory cytokines in plasma and hippocampal tissue (Data are presented as mean ± SD; *n* = 8; *∗∗P* < 0.01 *vs.* WT-Sham group; ^*##*^*P* < 0.01 *vs.* KO-Sham group; ^*&*^*P* < 0.05, ^*&&*^*P* < 0.01 *vs.* WT-II/R group).

Irisin is a factor cleaved from the type I membrane protein encoded by the *Fndc5* gene and subsequently released into the bloodstream. To further validate the role of irisin in GACD, we employed *Fndc5*^*−/−*^ mice to eliminate irisin expression (Fig. 4A). We first examined changes in FNDC5 expression in hippocampal tissue following II/R. The results revealed a progressive increase in FNDC5 expression within the first 48 h post-reperfusion, followed by a decline at 72 h (Fig. 4B and C). This temporal pattern was consistent with the trajectory of neuronal damage. Combined with the observed neuroprotective effects of irisin supplementation, we hypothesize that this upregulation of FNDC5 represents a compensatory protective mechanism. To further elucidate whether this change in FNDC5 level plays a protective role, we knocked out the *Fndc5* gene, which nearly abolished the expression of both FNDC5 and irisin (Fig. 4D–F). As anticipated, *Fndc5* knockout significantly exacerbated hippocampal injury following II/R (Fig. 4G and H). Additionally, *Fndc5*^*−/−*^ mice exhibited impaired spatial exploration and memory abilities compared to WT mice post-II/R (Fig. 4I–K). Elevated levels of inflammatory cytokines were observed in both plasma and hippocampal tissues of *Fndc5*^*−/−*^ mice compared to WT controls (Fig. 4L).

### 3.5 The reversal effect of irisin on GACD may be achieved by inhibiting TXINP to reduce the level of hippocampal neuroinflammation

**Fig. 5:**
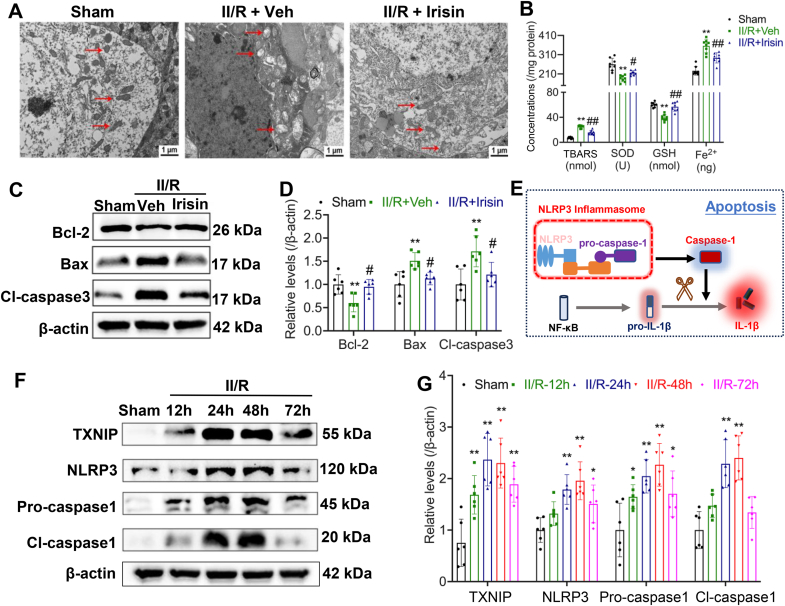
Irisin inhibits the inflammatory activation pathway in brain injury following II/R in mice. **(A)** Representative TEM images of hippocampal tissue (Red arrows indicate mitochondrial alterations within the different groups. Scale bar = 1 μm). **(B)** Quantification of indicators related to lipid peroxidation concentrations in the hippocampus after irisin treatment following II/R injury (*n* = 8; Data are presented as mean ± SD; ∗∗P < 0.01 vs. Sham group; #P < 0.05, ##P < 0.01 vs. Veh group). (C**)** Representative WB bands showing the levels of apoptosis-related molecules in the hippocampus after irisin treatment in II/R mice. β-actin was used as loading control and Bcl-2, Bax and Cl-caspase 3 correspond to a same loading controls **(D)** Relative levels of apoptosis-related molecules in the hippocampus (*n* = 6; Data are presented as mean ± SD; *∗∗P* < 0.01 *vs.* Sham group; ^*#*^*P* < 0.05 *vs.* Veh group). **(E)** Schematic diagram illustrating inflammasome assembly and activation. **(F)** Representative WB bands of FNDC5, TXNIP, NLRP3, Pro-caspase-1, and Cleaved caspase-1 in the hippocampus at different time points after II/R injury, -β-actin was used as loading control and all were referred to the same loading control. **(G)** Relative levels of FNDC5, TXNIP, NLRP3, Pro-caspase-1, and Cleaved caspase-1 in the hippocampus (*n* = 6; Data are presented as mean ± SD; *∗P* < 0.05, *∗∗P* < 0.01, *∗∗∗P* < 0.001 *vs.* Sham group).

To further elucidate the neuroprotective mechanisms of irisin in GACD, we first utilized TEM to examine the ultrastructural changes in brain tissue following II/R. TEM analysis revealed marked oxidative stress-induced damage, including mitochondrial swelling, structural impairment, and lipid peroxidation, all of which were significantly attenuated by irisin treatment (Fig. 5A). Based on these observations, we evaluated biomarkers associated with oxidative stress-induced ferroptosis. B-cell lymphoma 2 (Bcl 2) is an anti-apoptotic protein that inhibits programmed cell death by preventing mitochondrial outer membrane permeabilization. Bcl2-associated X protein (Bax) is a pro-apoptotic protein that promotes apoptosis by forming pores in the mitochondrial membrane, triggering cytochrome *c* release. The results indicated that irisin effectively reduced reactive oxygen species (ROS) levels and reversed the expression of ferroptosis-associated factors, as well as the degree of apoptotic damage (Fig. 5B–D). It is well-established that oxidative stress plays a pivotal role in activating and assembling the NLRP3 inflammasome. Given that TXNIP serves as an upstream regulator of NLRP3, we utilized Western blot analysis to assess changes in the TXNIP-NLRP3-mediated inflammatory signaling pathway. The results demonstrated a pronounced activation of the TXNIP-NLRP3 axis following II/R, with peak activation observed at 48 h post-reperfusion (Fig. 5E–G).

**Fig. 6:**
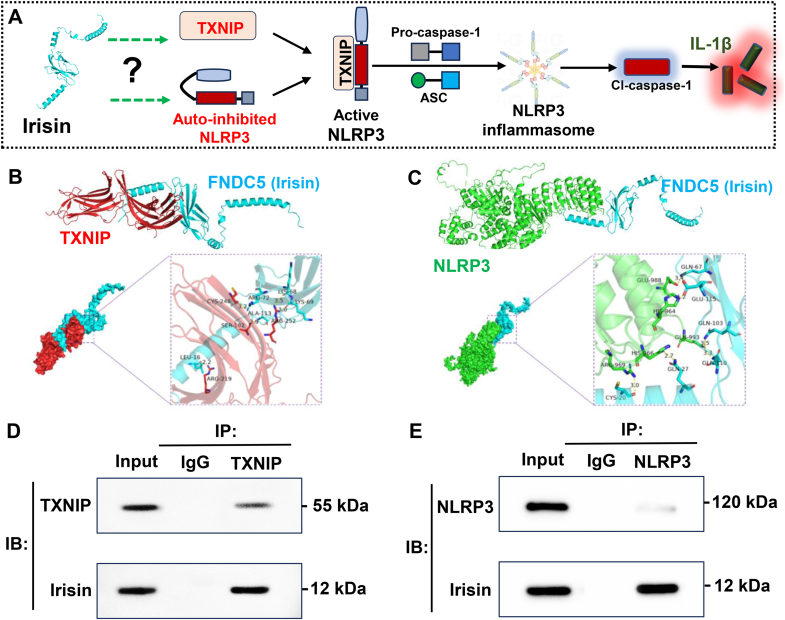
Interaction analysis of irisin with TXNIP and NLRP3 respectively. **(A)** Schematic diagram illustrating the TXNIP-NLRP3 axis. (B) Molecular interaction model of TXNIP with FNDC5/irisin. **(C)** Molecular interaction model of NLRP3 with FNDC5/irisin. **(D)** Co-IP representative bands for irisin and TXNIP interaction analysis. **(E)** Co-IP representative bands for irisin and NLRP3 interaction analysis.

**Fig. 7:**
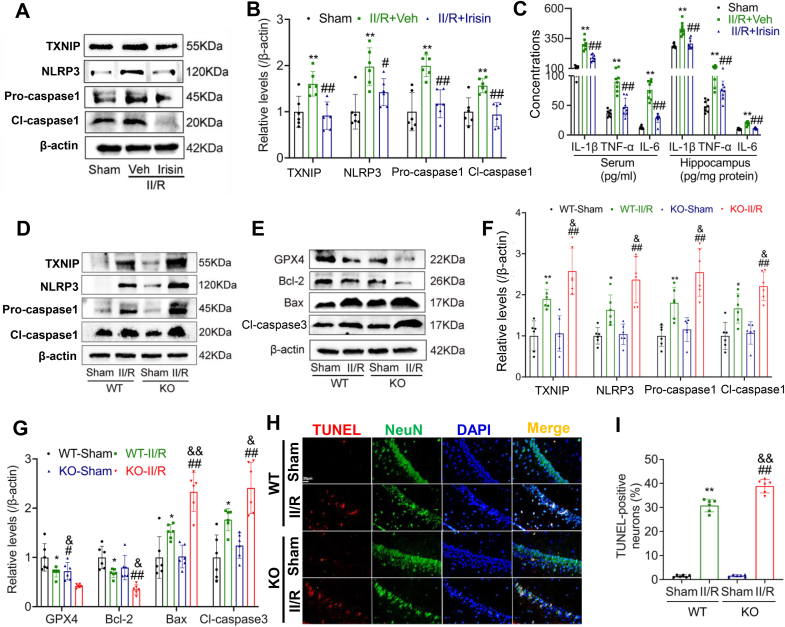
Irisin targets TXNIP to mitigate neuronal apoptosis in the hippocampus of II/R mice. **(A)** Representative WB bands of FNDC5, TXNIP, NLRP3, Pro-caspase-1, and Cleaved caspase-1 in the hippocampus of II/R mice after irisin treatment, β-actin was used as loading control and all were referred to the same loading control. **(B)** Relative levels of FNDC5, TXNIP, NLRP3, Pro-caspase-1, and Cleaved caspase-1 in the hippocampus (*n* = 6; Data are presented as mean ± SD; *∗∗P* < 0.01 *vs.* Sham group; ^*#*^*P* < 0.05, ^*##*^*P* < 0.01 *vs.* Veh group). **(C)** Concentrations of inflammatory cytokines in plasma and hippocampal tissue (*n* = 8; Data are presented as mean ± SD; *∗∗P* < 0.01 *vs.* Sham group; ^*##*^*P* < 0.01 *vs.* Veh group). **(D)** Representative WB bands of FNDC5, TXNIP, NLRP3, Pro-caspase-1, and Cleaved caspase-1 in the hippocampus of *Fndc5*-knockout II/R mice, β-actin was used as loading control and all were referred to the same loading control. **(E)** Representative WB bands of apoptosis-related molecules in the hippocampus of *Fndc5*-knockout II/R mice, β-actin was used as loading control and all were referred to the same loading control. **(F)** Relative levels of FNDC5, TXNIP, NLRP3, Pro-caspase-1, and Cleaved caspase-1 in the hippocampus (Data are presented as mean ± SD; *n* = 6; *∗P* < 0.05, *∗∗P* < 0.01 *vs.* WT-Sham group; ^*##*^*P* < 0.01 *vs.* KO-Sham group; ^*&*^*P* < 0.05 vs. WT-II/R group). **(G)** Relative levels of apoptosis-related molecules in the hippocampus (Data are presented as mean ± SD; *n* = 6; *∗P* < 0.05 *vs.* WT-Sham group; ^*##*^*P* < 0.01 vs. KO-Sham group; ^*&*^*P* < 0.05, ^*&&*^*P* < 0.01 vs. WT-II/R group). **(H)** Representative TUNEL staining images showing apoptotic neurons in the CA1 region of the hippocampus after FNDC5 knockout (Scale bar = 20 μm). **(I)** Quantification of the relative fluorescence intensity of TUNEL-positive cells in the CA1 region of the hippocampus (Data are presented as mean ± SD; *n* = 6; *∗∗P* < 0.01 *vs.* WT-Sham group; ^*##*^*P* < 0.01 *vs.* KO-Sham group; ^*&&*^*P* < 0.01 *vs.* WT-II/R group).

Building upon our findings regarding the neuroprotective effects of irisin, we hypothesize that its mechanism of action involves inhibition of inflammation-related signaling pathways. However, the specific target, whether TXNIP or NLRP3, remains to be determined. Protein interaction analyses revealed that FNDC5 exhibited a significantly higher binding affinity to TXNIP compared to NLRP3, with the formation of covalent bonds that likely suppress TXNIP function (Fig. 6A–C). See Supplementary Material 2 Figs. S2–3 for specific information on interconnection. Co-IP analysis revealed a strong interaction between irisin and TXNIP, whereas the association between irisin and NLRP3 was notably weak (Fig. 6D and E). Further investigations demonstrated that irisin supplementation markedly reduced TXNIP expression levels, subsequently attenuating downstream activation of the NLRP3 pathway and reducing levels of inflammatory cytokines (Fig. 7A–C). Conversely, FNDC5 knockout exacerbated activation of the TXNIP-NLRP3 pathway following II/R, accompanied by significant increases in oxidative stress-induced ferroptosis and apoptosis (Fig. 7D–I).

### 3.6 Irisin reduced the degree of neuroinflammation and promote injury repair by reducing the expression of TXNIP in microglia

**Fig. 8:**
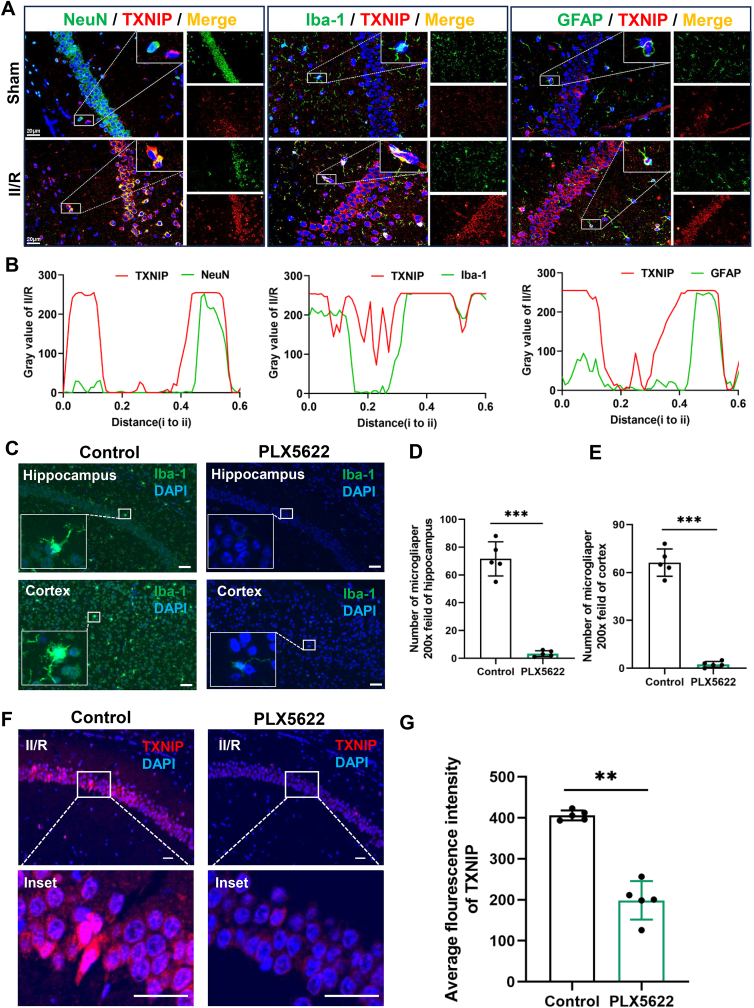
TXNIP may be mainly derived from microglia. **(A)** Co-localization of TXNIP with neurons, microglia, and astrocytes in the hippocampus (Scale bar = 20 μm). **(B)** Fluorescence co-localization distribution curves of TXNIP with NeuN, Iba-1, and GFAP, respectively. **(C)** Microglial depletion efficiency in mouse brain by PLX5622 treatment, showing Iba-1^+^ microglia (green) and DAPI^+^ nuclei (blue). Scale bar = 50 μm. **(D)** Quantitative analysis of microglial cell numbers in the hippocampus. **(E)** Quantitative analysis of microglial cell numbers in the cortex. **(F)** Effect of PLX5622-mediated microglial depletion on TXNIP level (red) in I/R mouse brain, with DAPI + nuclei (blue). Scale bar = 50 μm. **(G)** Analysis of TXNIP mean fluorescence intensity in II/R mouse brain. (Data presented as mean ± SEM; *∗∗P* < 0.01, *∗∗∗P* < 0.001 *vs.* control group).

To further clarify the cellular localization of irisin's target, we performed co-localization analyses of TXNIP with neurons, microglia, and astrocytes. Neuron specific marker NeuN, microglia marker ionized calcium-binding adapter molecule 1 (Iba-1) and astrocyte marker glial fibrillary acidic protein (GFAP) were used to show different cell locations, respectively. The results showed that TXNIP expression, which increased following II/R injury, was predominantly localized to microglia. TXNIP signals were also detected in neurons and astrocytes, but the co-localization signals were weaker than those in microglia. We speculate that TXNIP in II/R is primarily derived from microglia (Fig. 8A and B). To further determine whether microglia serve as the primary source of central TXNIP in II/R mice, we selectively depleted microglia using PLX5622 prior to establishing the II/R model. Results demonstrated that microglial ablation significantly reduced TXNIP expression in the hippocampus of II/R mice (Fig. 8C–G). These findings suggest that microglia-derived TXNIP represents a key target through which irisin exerts its neuroprotective effects.

**Fig. 9:**
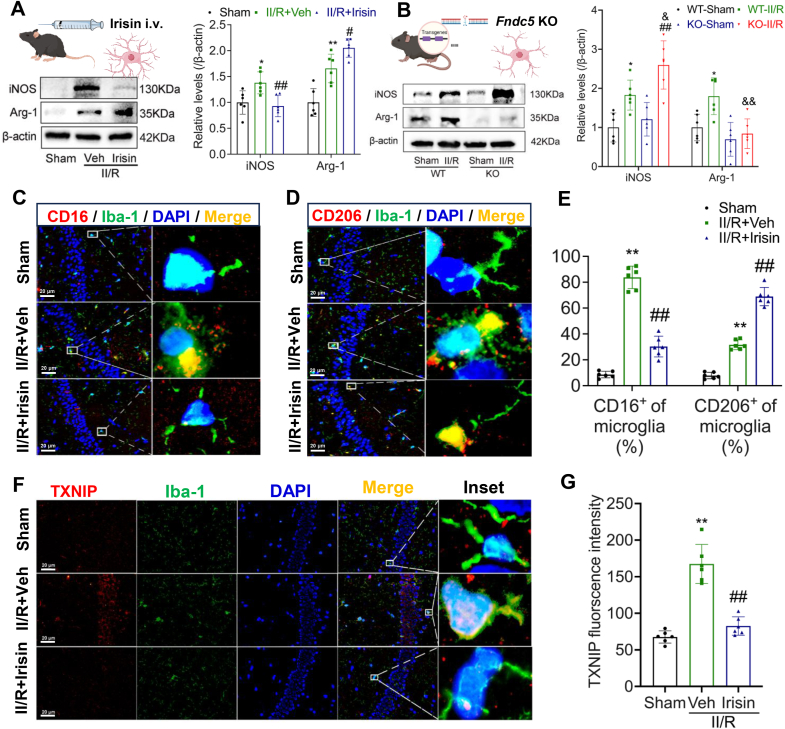
Irisin modulates microglial polarization by targeting TXNIP in microglia. **(A)** Representative WB bands and relative levels of iNOS and Arg-1 in the hippocampus of II/R mice after irisin treatment (β-actin was used as loading control and all were referred to the same loading control**,***n* = 6. Data are presented as mean ± SD; *∗P* < 0.05, *∗∗P* < 0.01 *vs.* Sham group; ^*#*^*P* < 0.05, ^*##*^*P* < 0.01 *vs.* Veh group). **(B)** Representative WB bands and relative levels of iNOS and Arg-1 in the hippocampus of *Fndc5*-knockout II/R mice (β-actin was used as loading control and all were referred to the same loading control. Data are presented as mean ± SD; *n* = 6; *∗P* < 0.05 *vs.* WT-Sham group; ^*##*^*P* < 0.01 *vs.* KO-Sham group; ^*&*^*P* < 0.05, ^*&&*^*P* < 0.01 *vs.* WT-II/R group). **(C**–**D)** Representative images showing the levels of M1 microglial marker CD16 and M2 microglial marker CD206 in the hippocampus (Scale bar = 20 μm). **(E)** Relative levels of CD16^+^ microglia and CD206^+^ microglia in the hippocampus (*n* = 6; Data are presented as mean ± SD; *∗∗P* < 0.01 *vs.* Sham group; ^*##*^*P* < 0.01 *vs.* Veh group). **(F)** Representative images of TXNIP co-localized with Iba-1 in the hippocampus after irisin treatment (Scale bar = 20 μm). **(G)** Relative fluorescence intensity of TXNIP level in the hippocampus (*n* = 6; Data are presented as mean ± SD; *∗∗P* < 0.01 *vs.* Sham group; ^*##*^*P* < 0.01 *vs.* Veh group).

Intravenous administration of recombinant irisin reduced the activation of pro-inflammatory microglia while enhancing the activation of reparative microglia, II/R mice exhibited a significant increase in the marker molecule inducible nitric oxide synthase (iNOS) for M1 microglia and a decrease in the marker molecule arginase-1 (Arg-1) for M2 microglia in the brain, which was partially rescued by irisin treatment (Fig. 9A and B). These findings were further validated using immunofluorescence, CD16 was used to label M1 microglia, and CD206 was used to label M2 microglia (Fig. 9C–G).

**Fig. 10:**
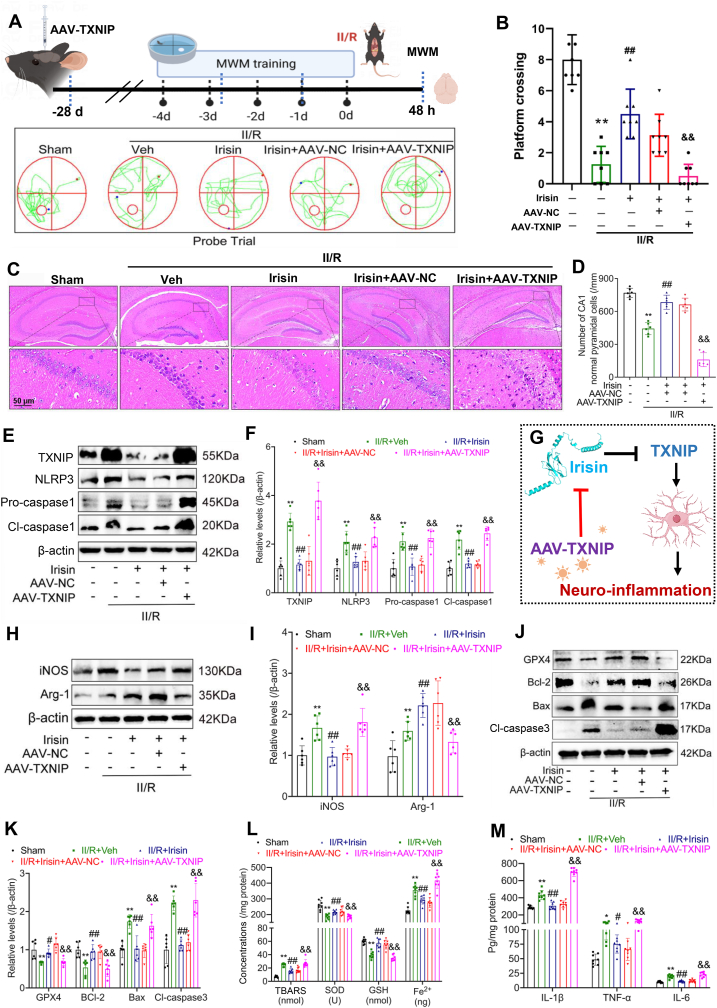
Overexpression of TXNIP reverses the neuroprotective effects of irisin. **(A)** Representative trajectory maps from the MWM test. **(B)** Number of platform crossings during the probe trial (Data are presented as mean ± SD; *n* = 6; *∗∗P* < 0.01, ^*##*^*P* < 0.01, ^*&&*^*P* < 0.01). **(C)** Representative H&E staining images of the hippocampal CA1 region 48 h after II/R injury (*n* = 6; scale bars = 50 μm). **(D)** Quantification of normal neurons in the hippocampal CA1 region (Data are presented as mean ± SD; *n* = 6; *∗∗P* < 0.01, ^*##*^*P* < 0.01, ^*&&*^*P* < 0.01). **(E)** WB analysis of FNDC5, TXNIP, NLRP3, pro-caspase-1, and cleaved caspase-1 levels in the hippocampus of II/R mice after TXNIP overexpression. β-actin was used as loading control and all were referred to the same loading control. **(F)** Relative protein levels of FNDC5, TXNIP, NLRP3, pro-caspase-1, and cleaved caspase-1 in the hippocampus (Data are presented as mean ± SD; *n* = 6; *∗∗P* < 0.01, ^*##*^*P* < 0.01, ^*&&*^*P* < 0.01). **(G)** Schematic representation of the proposed mechanism. **(H)** WB analysis the levels of iNOS and Arg-1 in the hippocampus of II/R mice following TXNIP overexpression, β-actin was used as loading control and all were referred to the same loading control. **(I)** Relative protein levels of iNOS and Arg-1 in the hippocampus (β-actin was used as loading control and all were referred to the same loading control. Data are presented as mean ± SD; *n* = 6; *∗∗P* < 0.01, ^*##*^*P* < 0.01, ^*&&*^*P* < 0.01). **(J)** WB analysis of apoptosis-related molecules in the hippocampus of II/R mice following TXNIP overexpression. β-actin was used as loading control and all were referred to the same loading control. **(K)** Relative protein levels of apoptosis-related molecules in the hippocampus (Data are presented as mean ± SD; *n* = 6; *∗∗P* < 0.01, ^*#*^*P* < 0.05, ^*##*^*P* < 0.01, ^*&&*^*P* < 0.01). **(L)** The concentrations of indicators related to lipid peroxidation in the hippocampus (Data are presented as mean ± SD; *n* = 6; *∗∗P* < 0.01, ^*##*^*P* < 0.01, ^*&&*^*P* < 0.01). **(M)** Quantitative analysis of pro-inflammatory cytokines concentrations in the hippocampus (Data are presented as mean ± SD; *n* = 6; *∗∗P* < 0.01, ^*#*^*P* < 0.05, ^##^*P* < 0.01, ^*&&*^*P* < 0.01).

We observed that TXNIP was localized predominantly in microglia. To further investigate whether TXNIP mediates the neuroprotective effects of irisin in GACD, we employed adeno-associated virus (AAV) to overexpress TXNIP specifically in the hippocampus of WT mice. The results indicated that TXNIP overexpression significantly reversed the neuroprotective effects of irisin. Specifically, following TXNIP overexpression, irisin failed to improve spatial memory performance in II/R mice (Fig. 10A and B) and lost its ability to attenuate neuronal inflammatory damage, inflammasome activation, microglial activation, ferroptosis, and neuroinflammation-related markers (Fig. 10C–M).

### 3.7 Irisin regulates microglial polarization through TXNIP thereby reducing neuronal damage

**Fig. 11:**
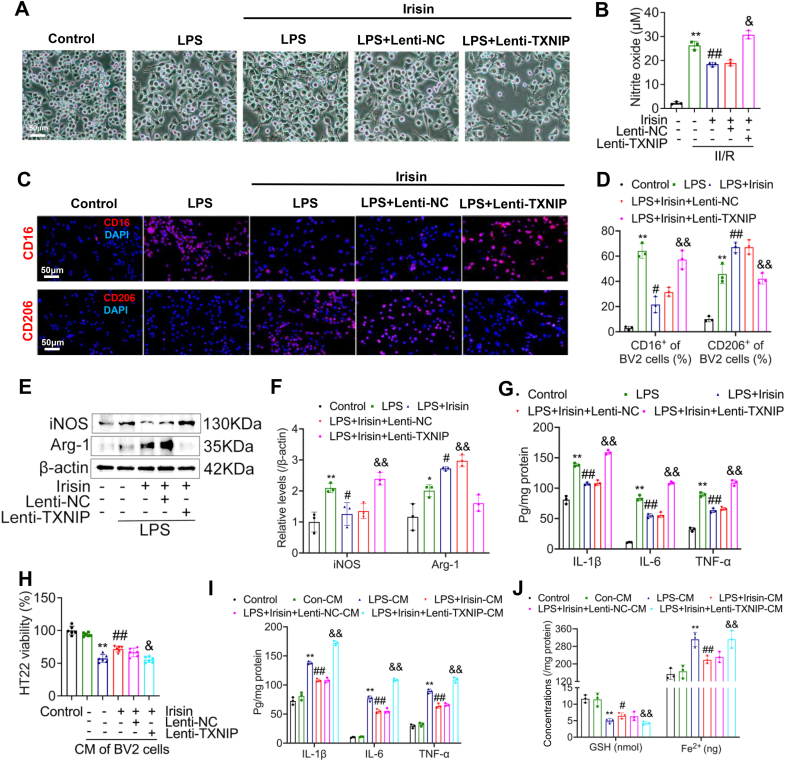
*In vitro* cell experiments confirm that irisin regulates microglial polarization via TXNIP. **(A)** Representative images of BV2 cells *in vitro* (Scale bar = 50 μm). **(B)** Nitric oxide (NO) levels in the culture medium of BV2 cells (Data are presented as mean ± SD; *n* = 3; *∗∗P* < 0.01 *vs.* Control group; ^*##*^*P* < 0.01 *vs.* LPS group; ^*&*^*P* < 0.05 *vs.* LPS + irisin group). **(C)** Representative fluorescence images of microglia *in vitro* showing markers for M1-and M2-polarized microglia (Scale bar = 50 μm). **(D)** Relative levels of CD16^+^ (M1) and CD206^+^ (M2) microglia *in vitro* (Data are presented as mean ± SD; *n* = 3; *∗∗P* < 0.01 *vs.* Control group; ^*#*^*P* < 0.05, ^*##*^*P* < 0.01 *vs.* LPS group; ^*&&*^*P* < 0.01 *vs.* LPS + irisin group). **(E)** Representative WB bands for iNOS and Arg-1. β-actin was used as loading control and all were referred to the same loading control. **(F)** Relative protein levels of iNOS and Arg-1 in the hippocampus (Data are presented as mean ± SD; *n* = 3; *∗P* < 0.05, *∗∗P* < 0.01 *vs.* Control group; ^*#*^*P* < 0.05 vs. LPS group; ^*&&*^*P* < 0.01 vs. LPS + irisin group). **(G)** Concentrations of pro-inflammatory cytokines IL-1β, IL-6, and TNF-α in the microglial culture medium (Data are presented as mean ± SD; *n* = 3; *∗∗P* < 0.01 *vs.* Control group; ^*##*^*P* < 0.01 *vs.* LPS group; ^*&&*^*P* < 0.01 *vs.* LPS + irisin group). **(H)** Cell viability of HT22 neurons co-cultured with microglia (Data are presented as mean ± SD; *n* = 6; *∗∗P* < 0.01 *vs.* Control group; ^*##*^*P* < 0.01 *vs.* LPS group). **(I)** Levels of inflammatory cytokines in the co-culture medium (Data are presented as mean ± SD; *n* = 3; *∗∗P* < 0.01 *vs.* Control group; ^*##*^*P* < 0.01 *vs.* LPS group; ^*&&*^*P* < 0.01 vs. LPS + irisin group). **(J)** Concentrations of glutathione (GSH) and ferrous iron (Fe^2+^) in the co-culture medium (Data are presented as mean ± SD; *n* = 3; *∗∗P* < 0.01 *vs.* Control group; ^*#*^*P* < 0.05, ^*##*^*P* < 0.01 *vs.* LPS group; ^*&&*^*P* < 0.01 *vs.* LPS + irisin group).

We cultured microglial cells *in vitro* to further elucidate whether irisin regulates microglial polarization through TXNIP. To achieve this, we overexpressed TXNIP in the BV2 microglial cell line. The results showed that, morphologically, LPS stimulation induced activation of microglia, characterized by an increase in cell body size. Treatment with irisin partially inhibited microglial activation, whereas overexpression of TXNIP abolished the protective effects of irisin, leading to a more pronounced activated state and elevated levels of nitric oxide (Fig. 11A and B). Further analysis of microglial activation markers revealed that irisin significantly increased the expression of CD206 while suppressing the expression of CD16. Overexpression of TXNIP reversed this effect (Fig. 11C and D). These findings were confirmed by Western blot analysis (Fig. 11E–G).

We co-cultured BV2 microglial cells and HT22 neuronal cells to further investigate whether irisin treatment and TXNIP overexpression directly influence neuronal damage induced by microglial activation. The results indicated that activated microglial cells significantly reduced neuronal cell viability. Treatment with irisin partially ameliorated this damage; however, this protective effect was abolished upon TXNIP overexpression. Furthermore, TXNIP overexpression led to elevated levels of inflammatory cytokines and ferroptosis-related molecules in the co-culture medium (Fig. 11H–J). These findings were further validated by oxidants react with DHE, flow cytometry, and TUNEL assays (Supplementary Material 2 Fig. S4).

## 4 Discussion

In the present study, we demonstrated the roles of FNDC5/irisin in GACD following II/R, and the potential mechanisms. II/R injury triggers systemic multi-organ dysfunction [29]. As demonstrated by our findings of muscle fiber atrophy and functional decline post-II/R. This muscle injury may disrupt the release of protective myokines, such as irisin, a muscle-derived factor cleaved from FNDC5. Our clinical data revealed a strong correlation between preoperative muscle thickness, plasma irisin levels, and cognitive outcomes in CPB patients, suggesting a potential gut-muscle-brain axis in II/R-related cognitive impairment. Notably, irisin supplementation attenuated hippocampal neuronal apoptosis, oxidative stress, and neuroinflammation in II/R mice, while *Fndc5* knockout exacerbated these injuries. These results highlight irisin's neuroprotective role in mitigating II/R-induced cerebral dysfunction, possibly through modulating microglial activation and downstream inflammatory pathways.

Our findings suggested that FNDC5/irisin contributed to the improvements in cerebral injury and cognitive deficits mediated by microglia after II/R. In line with the previous research, the aberrant increase of FNDC5/irisin in the damaged hippocampus may be attributed to the compensatory elevation of FNDC5/irisin and the transfer of FNDC5/irisin in circulation to counteract damage post-injury [30]. However, irisin has a relatively short tissue half-life and is exclusively derived from muscle tissue [31]. As muscle damage progresses, its production becomes significantly suppressed. Consequently, irisin transport to the brain inevitably declines with prolonged disease duration. Our findings highlight the critical role of muscle-derived factors in mediating brain injury following II/R. FNDC5 expression is induced not only by exercise but also appears to be modulated by muscle injury, as our study revealed that II/R leads to significant skeletal muscle damage, which may alter irisin production. This muscle-derived irisin plays an active neuroprotective role in the gut-brain axis by activating neuroprotective genes and exerting beneficial effects, including enhanced hippocampal blood flow, reduced neuroinflammation, attenuated oxidative stress, and improved memory and learning. Our findings suggest that muscle serves as a critical mediator in II/R-induced brain injury, where its dysfunction may exacerbate cognitive impairment, while preserving or restoring irisin levels could mitigate these effects. [[32], [33], [34]]. Skeletal muscle thickness reflects overall frailty in patients [35], and we found that quadriceps muscle depth was inversively correlated with postoperative certification impairment in CPB patients, further suggesting that muscle-derived bioactive substances are involved in brain protection after II/R. We specifically knockout FNDC5/irisin in mice, and showed that II/R in *Fndc5* ^*−/−*^ mice worsened pathologic alternations of the hippocampus and cognitive deficit, accompanied by graver neuroinflammation, oxidative stress, neuronal loss, including apoptosis and ferroptosis. Conversely, FNDC5/irisin supplement significantly improved the II/R-induced abnormal changes. These findings provide direct evidence that FNDC5/irisin contributed to the improvements of neuroinflammation, oxidative stress, and neuronal loss, which were involved in cerebral injury and cognitive deficits.

Besides, FNDC5/irisin-mediated microglial activation has been reported to be involved in cerebral ischemia and intracerebral hemorrhage, and it ameliorated neurological deficits, neuroinflammation, oxidative stress, and neuronal apoptosis [12,36]. Overactive microglia can mediate oxidative stress and inflammatory cytokine release. Moreover, the oxidative stress and release of inflammatory cytokines may act as second messengers to amplify the microglial activation [37,38], forming a vicious positive feedback loop and finally accelerating the development of brain injury and memory deficits [39,40]. Hitherto, mechanisms underlying the microglial activation after II/R are yet unclear, and only limited data are available on the exact activation patterns of microglial by FNDC5/irisin [12,36]. In this study, we found that II/R induced the activation of microglia, especially M1. Moreover, FNDC5/irisin deficiency exacerbated M1 microglial activation and partially interrupted the M2 microglial activation, with aggravated neuroinflammation, oxidative stress, and neuronal loss. Recombinant FNDC5/irisin supplement precipitated microglial polarization from M1 to M2 after II/R, causing the inhibition of neuroinflammation, oxidative stress, and neuronal loss, which further improved cognitive deficits induced by II/R. Thus, our study revealed that through regulating microglial activation, FNDC5/irisin improved the II/R-induced neuroinflammation, oxidative stress, and neuronal loss, which further improved cognitive deficits, a newly discovered potential therapeutic target.

In this experiment, we discovered that II/R-induced neuroinflammation, oxidative stress, neuronal apoptosis and ferroptosis were accompanied by TXNIP expression in microglia. It has been reported that pathological neuroinflammation were associated with TXNIP in some central nervous system disorders, especially via the TXNIP/NLRP3 pathway [[41], [42], [43], [44]]. And the TXNIP/NLRP3 pathway also triggered neuronal apoptosis [[42], [43], [44], [45], [46], [47]]. According to recent researches, TXNIP participated in the ferroptosis of frucose-induced renal injury, diabetic retinopathy, and multiple sclerosis [[48], [49], [50], [51]]. And TXNIP was also identified as a ferroptosis-related gene in multiple tumor diseases [[52], [53], [54]]. Moreover, increasing studies have shown that TXNIP is closely correlated with microglial activation [55]. Consistent with the previous findings [[56], [57], [58]], the colocalization of TXNIP with microglia, neurons, and astrocytes was distinctly observed in this study. *Fndc5*^*−/−*^ mice showed a higher expression of the TXNIP and more M1 microglial activation as a response to II/R, while recombinant FNDC5/irisin inhibited the TXNIP, which was accompanied by the activation of M2 microglia. Herein, we identified an association between FNDC5/irisin, microglia, and TXNIP. Next, via overexpressing TXNIP in BV2 cells and the specific CA1 area of the hippocampus, the effect of FNDC5/irisin on microglia polarization was reversed, and the effects of FNDC5/irisin on neuroinflammation, oxidative stress, and neuronal loss, including apoptosis and ferroptosis, were interrupted at the same time. Finally, the improvement of the II/R-induced cognitive deficit and cerebral injury by FNDC5/irisin were abolished. These results indicated that the neuroprotective effects of FNDC5/irisin on II/R-induced hippocampal pathological alteration and cognitive deficit are exerted, at least partially, via inhibiting the TXNIP in microglia which further promoted microglial polarization from M1 to M2 phenotype.

Nevertheless, the present study has some limitation. Firstly, only the early effects of FNDC5/irisin deficiency were explored without further studying its long-term effects on II/R-mediated GACD. Secondly, this study only discussed the microglial activation-associated pro-inflammation, pro-oxidative stress, pro-ferroptosis, and pro-apoptosis effects of FNDC5/irisin deficiency; the other mechanisms need to be investigated further. We only indirectly proved the modulation between FNDC5/irisin and TXNIP; whether FNDC5/irisin directly acts on TXNIP is yet unclear. Thus, future studies need to address the correlation between them. Finally, it is also important to note that a variety of myokines derived from muscle may also be involved in II/R-related brain damage. The common myokines include interleukin-6 (IL-6), fibroblast growth factor 21 (FGF21), Follistatin, insulin-like growth factor 1 (IGF1), brain-derived neurotrophic factor (BDNF) and vascular endothelial growth factor (VEGF) [59], which may also have important roles in the nervous system, so further omics analysis may be needed to clarify the changes in various myokines. Additionally, it is worth noting that the cellular sources of TXNIP may vary across different diseases. In future studies, employing cell-specific knockout models or single-cell sequencing technologies could help further elucidate the origins and functional roles of TXNIP.

## 5 Conclusions

**Fig. 12:**
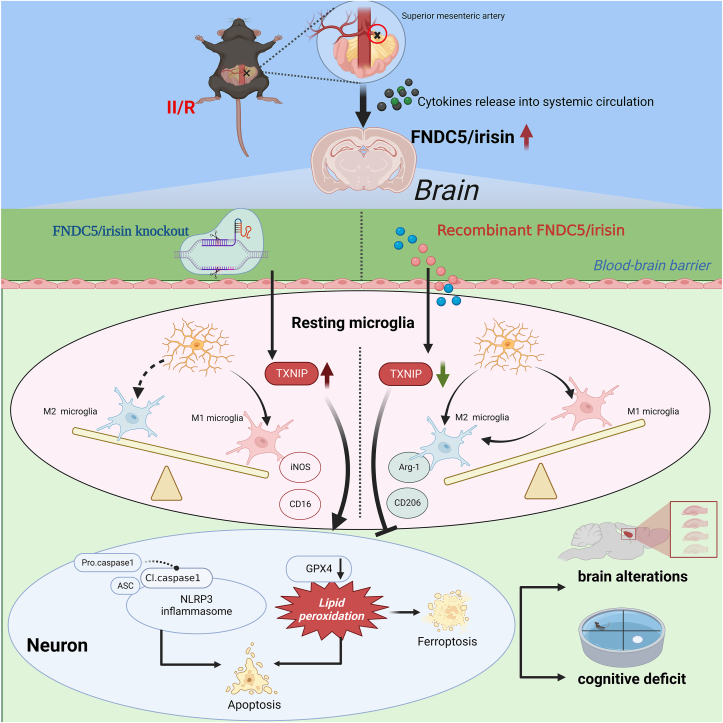
Schematic diagram depicting the mechanisms by which FNDC5/irisin–mediated microglial anti-inflammatory phenotype polarization, and further improved II/R-mediated brain injure and cognitive deficits via **the TXNIP.** TXNIP facilitates the interaction between FNDC5/irisin and microglia, which promotes the microglial anti-inflammation phenotype polarization, thus suppressing pro-inflammation phenotype polarization, resulting in the improvement of cognitive deficits and the inhibition of oxidative stress, neuroinflammation, and neuronal loss after II/R.

This study reveals that FNDC5/irisin protects against cognitive impairment after II/R injury by attenuating neuroinflammation. FNDC5/irisin deficiency worsens brain damage, while its supplementation preserves neuronal function. Mechanistically, FNDC5/irisin downregulates TXNIP in microglia, promoting a shift from pro-inflammatory M1 to anti-inflammatory M2 polarization and inhibiting NLRP3 inflammasome activation. The FNDC5/irisin-TXNIP axis is thus a key regulator of II/R-induced neuroinflammation (Fig. 12). These findings deepen our understanding of gut-muscle-brain crosstalk and position FNDC5/irisin as a potential therapy for II/R-related cognitive dysfunction.

## CRediT authorship contribution statement

**Yafang Tan:** Writing – original draft, Software, Methodology, Investigation, Formal analysis, Data curation. **Guo Mu:** Writing – review & editing, Writing – original draft, Funding acquisition, Conceptualization. **Feixiang Wang:** Writing – original draft, Software, Formal analysis, Data curation. **Xin Fan:** Methodology, Formal analysis, Data curation. **Chengjie Yang:** Investigation, Data curation. **Zuan Shi:** Data curation. **Yiping Bai:** Software, Resources, Data curation. **Bingqing Xie:** Funding acquisition, Conceptualization. **Xuan Yu:** Data curation, Conceptualization. **Jianguo Feng:** Validation, Resources, Data curation, Conceptualization. **Jing Jia:** Data curation. **Xiaobin Wang:** Visualization, Validation, Supervision. **Ye Chen:** Writing – original draft, Supervision, Funding acquisition, Conceptualization. **Jun Zhou:** Writing – review & editing, Writing – original draft, Visualization, Validation, Supervision, Project administration, Methodology, Investigation, Funding acquisition, Formal analysis, Data curation, Conceptualization.

## Funding

This study was supported by grants from the Sichuan Science and Technology Program (No.25NSFSC1924), Luzhou Science and Technology Program (No.2023SYF099), Zigong Key Science and Technology Plan (Collaborative Innovation Project of Zigong Institute of Brain Sciences, No.2024-NKX-01-02).

## Declaration of competing interest

The authors declare that they have no known competing financial interests or personal relationships that could have appeared to influence the work reported in this paper.

## Data Availability

Data will be made available on request.
