# Supplementary material for: Muscle-derived factor alleviated cognitive impairment caused by intestinal ischemia-reperfusion
Source: Redox Biol. 2025 May 15;84:103682. doi: 10.1016/j.redox.2025.103682 (PMC12145813; doi:10.1016/j.redox.2025.103682)
Supplement: Multimedia component 1 [file mmc1.docx]

**Supplementary Material 1**

| Gene | Primer sequences |
| --- | --- |
| FNDC5/irisin | Forward:CTGTCTCCAATGTTCCACTTGTCTG |
|  | Reverse1:CTTGCCTTTGTTCTTTGAGGCCATC |
|  | Reverse2:GCTTGAACCAAGGCGAGAGCTAGT |
| TXNIP | Forward: ATACTCCTTGCTGATCTACG |
|  | Reverse: TGGGGTATCTGGGATGTTTA |
| GAPDH | Forward: TGGCCTTCCGTGTTCCTAC |
|  | Reverse: GAGTTGCTGTTGAAGTCGCA |

**Supplementary Table S1.** Primer sequences used for qRT-PCR
