# Supplementary material for: Muscle-derived factor alleviated cognitive impairment caused by intestinal ischemia-reperfusion
Source: Redox Biol. 2025 May 15;84:103682. doi: 10.1016/j.redox.2025.103682 (PMC12145813; doi:10.1016/j.redox.2025.103682)
Supplement: Multimedia component 2 [file mmc2.docx]

**Supplementary Material 2**

**Figure S1**


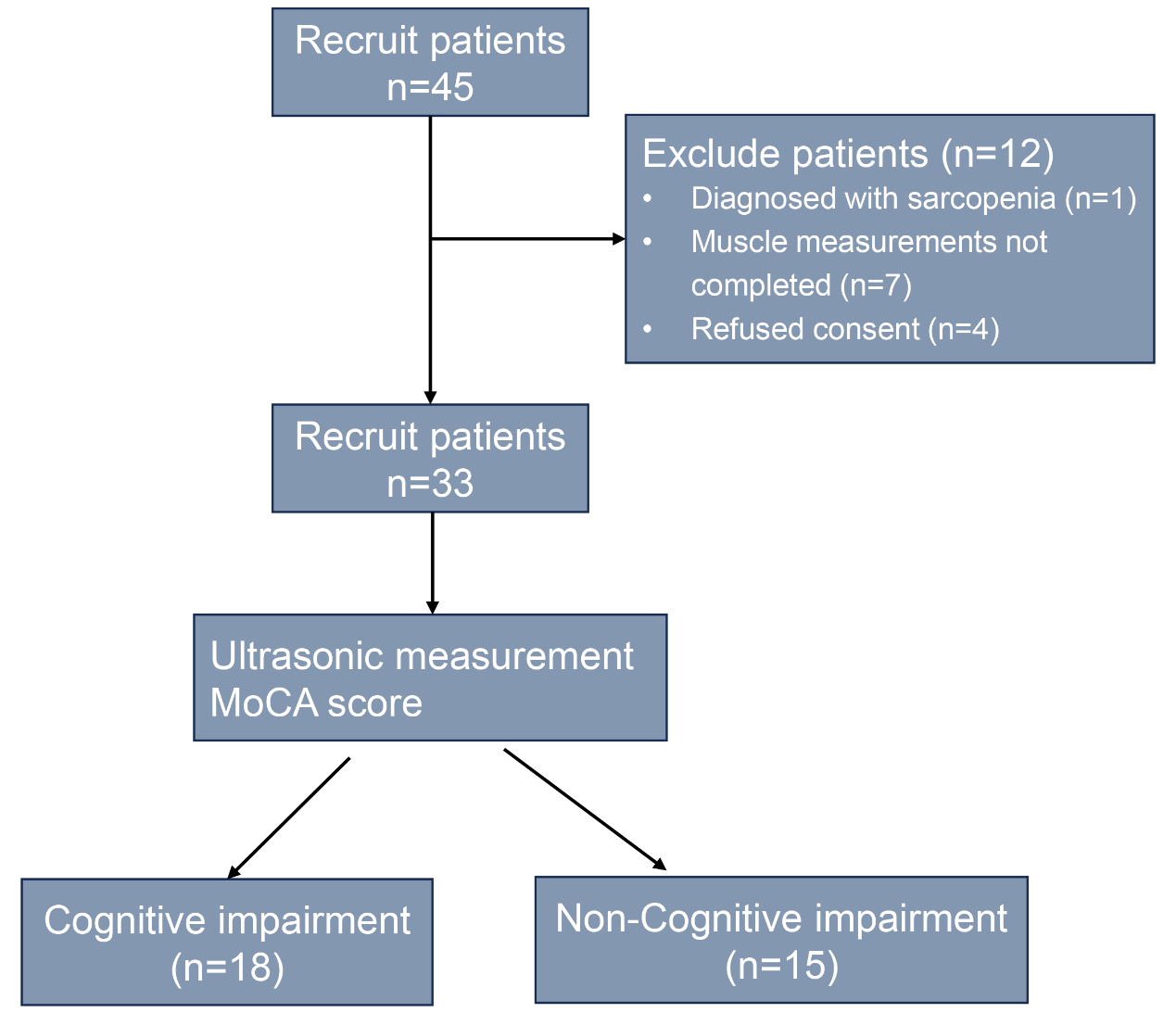


**Figure S1**. Patient inclusion and exclusion flow chart

**Figure S2**


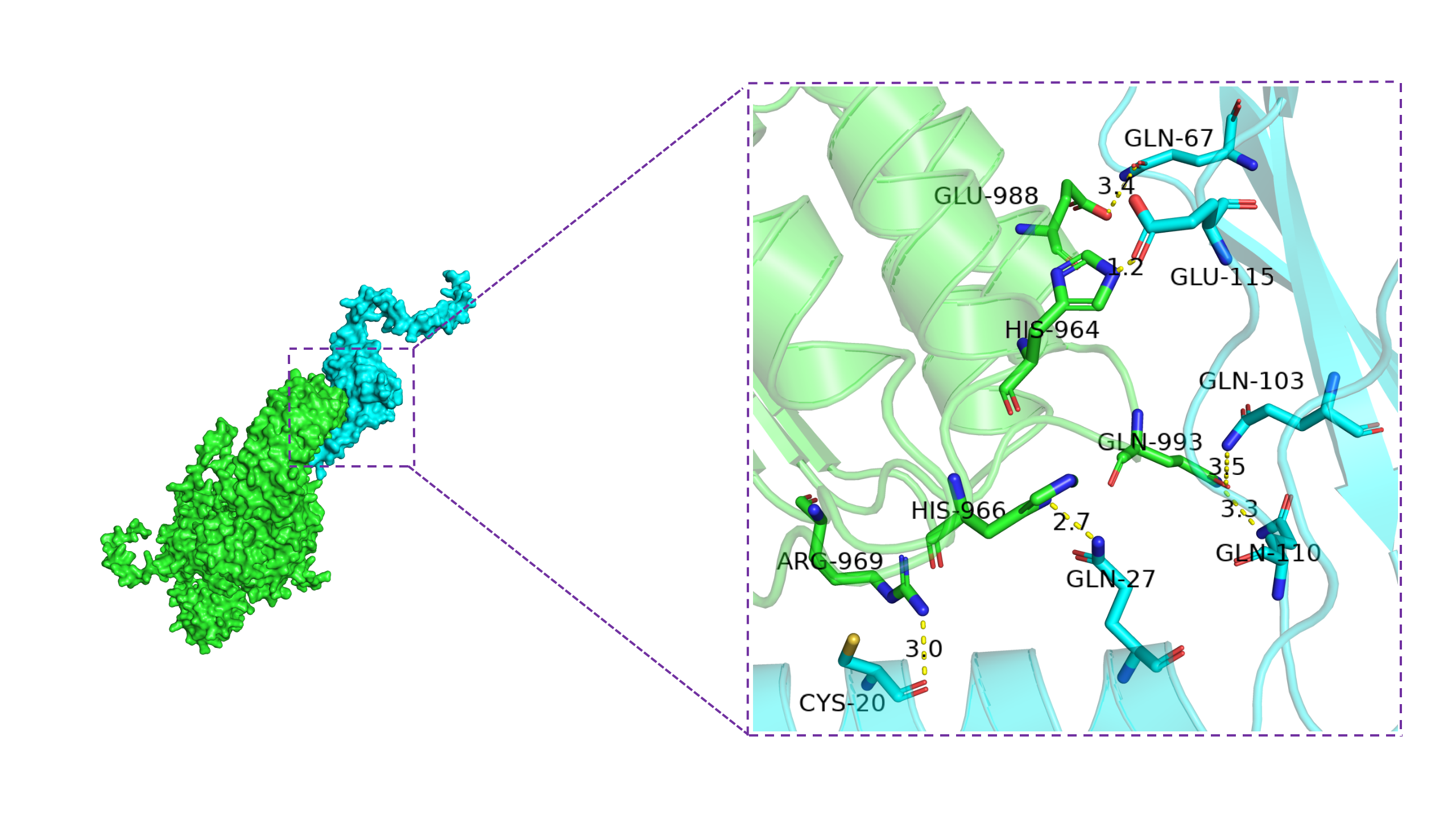


**Figure S2.** The results show that the binding energy of FNDC5 and NLRP3 is -324.11kcal/mol. Residues around protein-protein interaction interfaces can form hydrogen bonds. These non-covalent bonds can help stabilize protein-protein complexes. ARG-969, HIS-966, GLN-993, HIS-964 and GLU-988 of N form hydrogen bonds with CYS-20, GLN-27, GLN-110, GLN-103, GLU-115 and GLN-67 of Fndc5, respectively. The hydrogen bond lengths were 3.0 Å, 2.7 Å, 3.3 Å, 3.5 Å, 1.2 Å and 3.4 Å, respectively. The above residues may be the residues that play an active role in both.

**Figure S3**


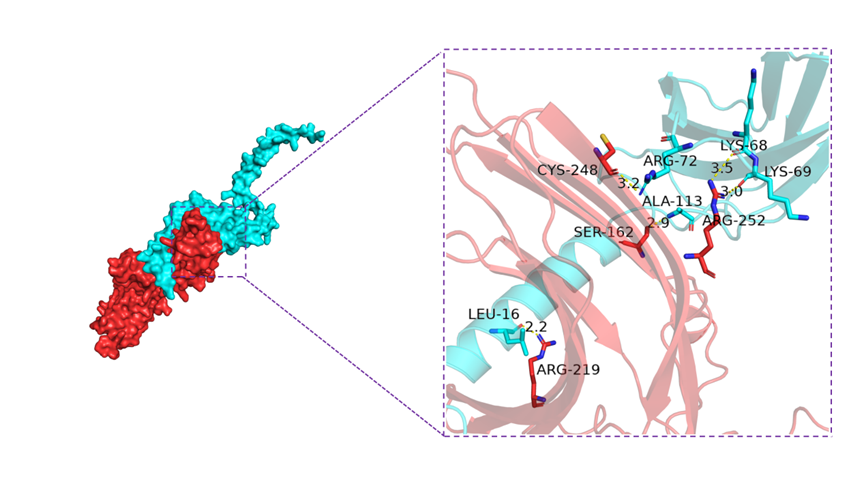


Figure S3. The results show that the binding energy of FNDC5 and TXNIP is -255.9kcal/mol. Residues around protein-protein interaction interfaces can form hydrogen bonds. These non-covalent bonds can help stabilize protein-protein complexes. ARG-219, ARG-252, SER-162 and CYS-248 of Txnip form hydrogen bonds with LEU-16, LYS-69, LYS-68, ALA-113 and ARG-72 of Fndc5, respectively. The hydrogen bond lengths were 2.2 Å, 2.9 Å, 3.0 Å, 3.5 Å and 3.2 Å, respectively. The above residues may be the residues that play an active role in both.

**Figure S4**


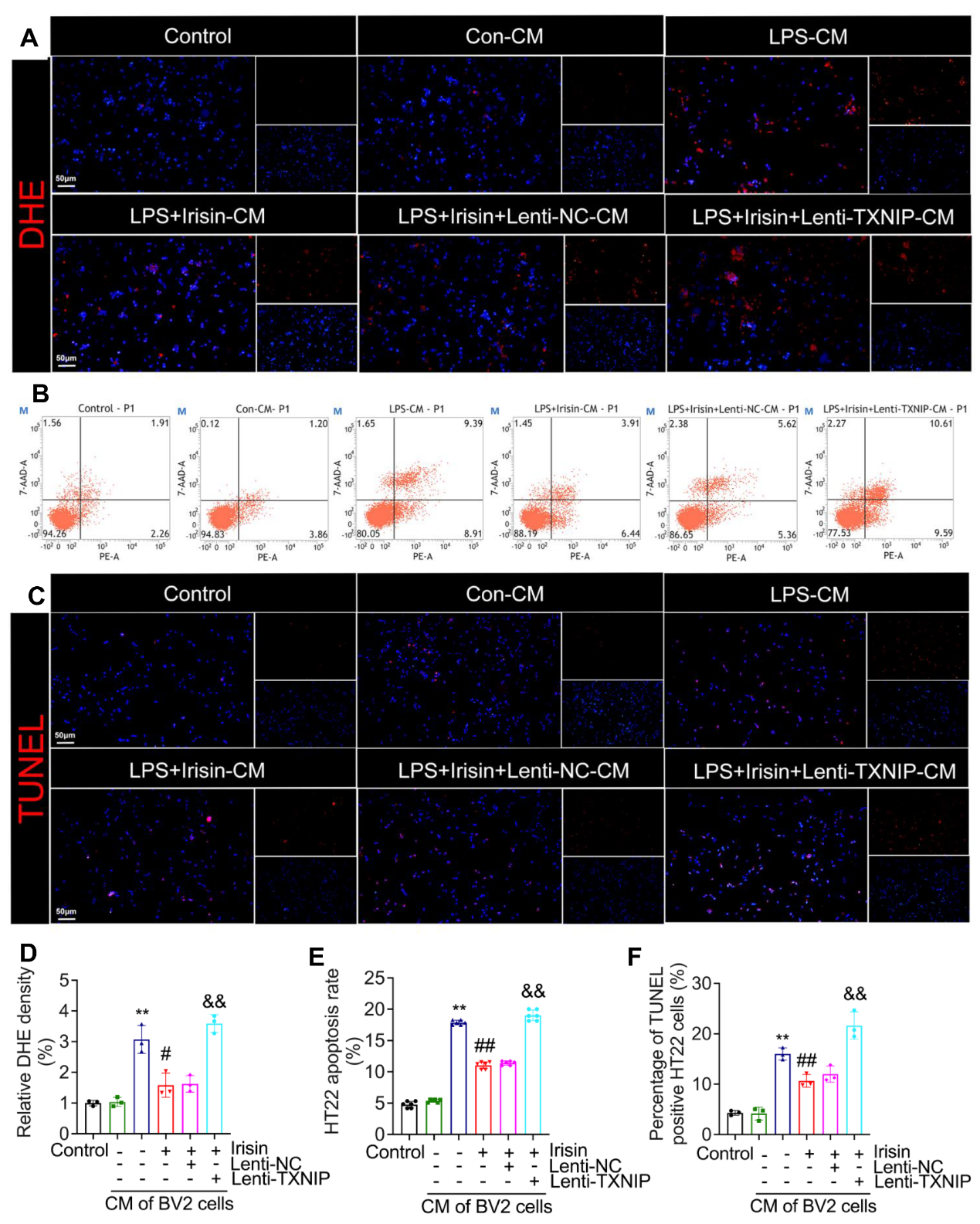


**Figure S4 TXNIP overexpression abolishes the effects of FNDC5/irisin on neurons *in vitro*.**

(A) Representative microphotographs of DHE staining of HT22 cells. (Magnification: ×200, scale bars = 50 μm). (B) Representative flow cytometry plots of HT22 cells. (C) Representative microphotographs of TUNEL-positive HT22 cells. (Magnification: ×200, scale bars = 50 μm). (D) Relative DHE density. (E) The proportion of apoptosis of HT22 cells was quantified by flow cytometry. (F)Percentage of TUNEL positive of HT22 cells. Data are represented as mean ± SD. *P < 0.05 vs. Control group; **P < 0.01 vs. Control group; #P < 0.05 vs. LPS group; ##P < 0.01 vs. LPS group. &P < 0.05 vs. LPS+ Irisin + Lenti-NC-CM group; &&P < 0.01 vs. LPS+ Irisin + Lenti-NC-CM group.
